# Microbial lag phase can be indicative of, or independent from, cellular stress

**DOI:** 10.1038/s41598-020-62552-4

**Published:** 2020-04-03

**Authors:** Philip G. Hamill, Andrew Stevenson, Phillip E. McMullan, James P. Williams, Abiann D. R. Lewis, Sudharsan S, Kath E. Stevenson, Keith D. Farnsworth, Galina Khroustalyova, Jon Y. Takemoto, John P. Quinn, Alexander Rapoport, John E. Hallsworth

**Affiliations:** 1 Institute for Global Food Security, School of Biological Sciences, Queen’s University Belfast, 19 Chlorine Gardens, Belfast, BT9 5DL Northern Ireland; 2 Department of Chemistry, PGP College of Arts and Science, NH-7, Karur Main Road, Paramathi, Namakkal, Tamil Nadu 637 207 India; 3 Special Collections and Archives, McClay Library, Queen’s University Belfast, 10 College Park Avenue, Belfast, BT7 1LP Northern Ireland; 4 Laboratory of Cell Biology, Institute of Microbiology and Biotechnology, University of Latvia, Jelgavas Str., 1-537, LV-1004 Riga, Latvia; 5 Utah State University, Department of Biology, 5305 Old Main Hill, Logan, UT 84322 USA

**Keywords:** Biotechnology, Microbiology

## Abstract

Measures of microbial growth, used as indicators of cellular stress, are sometimes quantified at a single time-point. In reality, these measurements are compound representations of length of lag, exponential growth-rate, and other factors. Here, we investigate whether length of lag phase can act as a proxy for stress, using  a number of model systems (*Aspergillus penicillioides*; *Bacillus subtilis*; *Escherichia coli*; *Eurotium amstelodami*, *E. echinulatum*, *E. halophilicum*, and E. repens; *Mrakia frigida*; *Saccharomyces cerevisiae*; *Xerochrysium xerophilum*; *Xeromyces bisporus*) exposed to mechanistically distinct types of cellular stress including low water activity, other solute-induced stresses, and dehydration-rehydration cycles. Lag phase was neither proportional to germination rate for *X. bisporus* (FRR3443) in glycerol-supplemented media (r^2^ = 0.012), nor to exponential growth-rates for other microbes. In some cases, growth-rates varied greatly with stressor concentration even when lag remained constant. By contrast, there were strong correlations for *B. subtilis* in media supplemented with polyethylene-glycol 6000 or 600 (r^2^ = 0.925 and 0.961), and for other microbial species. We also  analysed data from independent studies of food-spoilage fungi under glycerol stress (*Aspergillus aculeatinus* and *A. sclerotiicarbonariu*s); mesophilic/psychrotolerant bacteria under diverse, solute-induced stresses (*Brochothrix thermosphacta*, *Enterococcus faecalis*, *Pseudomonas fluorescens*, *Salmonella typhimurium*, *Staphylococcus aureus*); and fungal enzymes under acid-stress (*Terfezia claveryi* lipoxygenase and *Agaricus bisporus* tyrosinase). These datasets also exhibited diversity, with some strong- and moderate correlations between length of lag and exponential growth-rates; and sometimes none. In conclusion, lag phase is not  a reliable measure of stress because length of lag and growth-rate inhibition are sometimes highly correlated, and sometimes not at all.

## Introduction

Chemical reactions and thermodynamic and biological processes often experience a lag period prior to reaching their maximum rate. This phenomenon that can be observed at various levels; thermodynamic processes (e.g. thermal lag), chemical reactions, biochemical activities^1^, cellular physiology^2^, microbial growth kinetics, and  ecosystem functions^3^. The term *lag*, used in English since the early 14^th^ century, has been applied to biological processes since at least the 1680s^4^. In the context of microbial growth kinetics, lag, once known as ‘latency’, was studied since the 1800s, including work of Louis Pasteur^5^. Despite this long pedigree of research into this phenomenon, however, some aspects of the biology and application of the lag phase are not yet resolved.

Accurate assessments of microbial growth must account for the various growth processes, including: mycelial extension, cell division within planktonic populations, increases in the mass of individual cells, accumulation of compatible solutes or other endogenous reserves, cell elongation or growth, sporulation, germination, biofilm development, other forms of differentiation (e.g., primordium- and fruiting-body formation), and invasion and colonization of new habitats. Regardless of this, a phase of zero- or near-zero growth commonly precedes exponential growth. The duration of this ‘lag’ phase is by definition  slightly less than that of the period preceding exponential growth (see below). During early studies, lag phase was quantified by different means^6^, but is usually now determined according to the method of Lodge and Hinshelwood (1943)^7^.

Microbial cells are active during all or most of the lag phase, and the physiological and molecular changes during this period have been well-characterized^8–10^. Lag-phase metabolism may include the activation of signalling pathways^11^ and transcriptional changes leading to the upregulation of protein assembly, nucleotide metabolism, lipopolysaccharide biosynthesis, respiration, and other processes which are needed for differentiation and multiplication^12^. Collectively, such activities lead to cell division, and can facilitate exponential growth.

In relation to both laboratory-based experiments and ecological studies carried out *in situ*, lag phase may impact the outcomes of studies of stress tolerance, biomass yield, habitability, vigour, competitive ability, and other parameters. Cellular stress occurs in all types of microbial system; furthermore, microbes can  never be entirely  stress-free^13^. There is an element of autonomy in relation to essential processes that occur prior to germination proper, the processes of breakdown of compatible solutes and RNA degradation^14^. This said, cells may also experience environmental challenges that necessitate more-extensive periods of adjustment before growth can commence and so lengthen the lag phase. These can include various (mechanistically distinct) stresses. By way of example, a minimal lag phase for germination of conidia may span several hours, whereas stressors and/or temperature may lengthen this to days, months, or even longer^15–17^. The same stress parameter(s) may also constrain rates of cell division if and when exponential growth commences^18,19^. In other words, cellular stress may sometimes prolong lag-phase and then also cause low rates of exponential growth for a given microbial population^19,20^.

Single time-point determinations of optical density, cell number, dry weight and fresh biomass are therefore used to quantify stress for diverse microbial systems^21–27^. The same methodology is also used during studies of stress in plant species^28–31^. However, measurements obtained this way actually represent multiple parameters such as lag phase, size of inoculum, the proportion of viable cells, heterogeneity of growth rates within the population, and the rates of subsequent (exponential) growth. Lag phase is often assumed to correlate with cellular stress^32–37^ and has even been used as a direct measure of product-induced inhibition of growth^38–40^. Historically, cellular stress has most-commonly been defined according to empirically determined constraints on exponential growth rate^25,41–46^. During exponential growth under biophysical or physicochemical constraints, growth rate acts as a direct measure of stress on the system^13^. The validity of using single time-point determinations of biomass, colony formation or germination to quantify stress, however, depends on whether lag phase is proportional to the rate of exponential growth. Thus far, there is a paucity of information on whether lag phase is a meaningful as a  proxy for stress for different types of growth phenotype (e.g. planktonic cell division versus radial growth of filamentous colonies), different phylogenetic taxa, and/or mechanistically distinct types of cellular stress.

The current study was carried out to test whether length of the microbial lag phase is a reliable measure of cellular stress. All microbes are aqueous systems that function in essentially aqueous environments, so the primary type of stress that can impact all microbial life is solute-induced stress. Solutes cause stress in microbial cells via various mechanisms: e.g. water activity reduction, osmotically (turgor-induced changes), and/or chaotropicity^47^. Hyper-osmotic stress dehydrates cells, and chaotropicity can cause lipid peroxidation which, in turn, induces oxidative damage^42^. Solute-induced and water-related stresses, arguably the most-potent that shape ecosystem and biosphere function, were therefore used as the basis for the study system. We used a range of microbial systems (and enzymes from two microbes) as models from  different domains of life, that encompassed different processes (growth of hyphal cultures and planktonic cultures, germination of propagules), and ecophysiological distinct groups (e.g. mesophiles, xerophiles) (Table S1). The models were: spore germination of fungal xerophiles (12 strains) at extreme water-activities (0.741 to 0.585), *Saccharomyces cerevisiae* subjected to dehydration-rehydration stress, planktonic growth of *Bacillus subtilis* and *Escherichia coli*, and colony formation of the  psychrophilic yeast *M. frigida* under mechanistically diverse solute-induced stresses. The fungal xerophiles *A. penicillioides, Eurotium amstelodami, Eurotium echinulatum, Eurotium halophilicum, Eurotium repens, Xerochrysium xerophilum* and *Xeromyces bisporus* represent the extreme fringe of the microbial biosphere in terms of their ability to function at low water-activity^48,49^; *S. cerevisiae* is important for bioethanol production and in food and other applications; the soil bacterium *B. subtillis* is an important research model and factory of choice for various white biotechnologies; and *E. coli* is a pathogen and important research model. We also analysed datasets from independent studies of microbial and enzyme systems: mycelial extension of mesophilic fungi (*Aspergillus aculeatinus* and *Aspergillus sclerotiicarbonarius* under glycerol stress), planktonic growth of mesophilic and psychrotolerant bacteria (*Enterococcus faecalis*, *Salmonella typhimurium* and *Staphylococcus aureus*, and *Brochothrix thermosphacta* and *Pseudomonas fluorescens*, respectively, under glycerol-, NaCl- and sucrose-induced stresses); and catalytic activity of fungal lipoxygenase and tyrosinase under acid stress. These were selected on the basis that *Aspergillus aculeatinus*, *A. sclerotiicarbonarius, B. thermosphacta*, *E. faecalis*, *P. fluorescens, S. typhimurium* and *S. aureus* are food-spoilage microbes; and the enzyme systems which are pertinent to food quality and/or development of human diseases. Each of the microbes used in the experiments, and those used for meta-analyses, can be exposed to dehydration-rehydration and/or extreme, solute-induced stresses in their natural habitats and/or anthropogenic systems. The specific aims of the current study were to: (i) determine windows of tolerance to solute- and water-induced stresses; (ii) determine whether there is a relationship between length of lag phase and exponential growth rates under these stresses for diverse model microbes; and (iii) consider the implications for studies of microbial stress biology.

## Results and discussion

### Windows of stress tolerance

Whereas some types of extremophilic Bacteria and Archaea are more stress-tolerant than their eukaryote counterparts, fungi are generally more xerophilic than prokaryotes, with the exception of extreme bacterial and archaeal halophiles^17,18,50^. The biotic windows for growth and germination of model microbes used for experiments in the current study are  shown in Table S2. Xerophile germination occurred at the lowest water-activity (0.637) on malt extract yeast extract phosphate agar (MYPiA) supplemented with glycerol +sucrose, for *X. bisporus* strains (Fig. S1; Table S3). The most-xerophilic fungus known, however, is *A. penicillioides* strain JH06THJ which germinated down to 0.640 water activity in the current study (Fig. S1; Table S3), but at water activities down to 0.585 under other environmental conditions^17^. Water-activity reduction is the primary mechanism by which glycerol induces cellular stress at relatively moderate concentrations^47^, but chaotropicity becomes the limiting parameter when glycerol is present at concentrations of > 5 M^51,52^.

**Figure 1:**
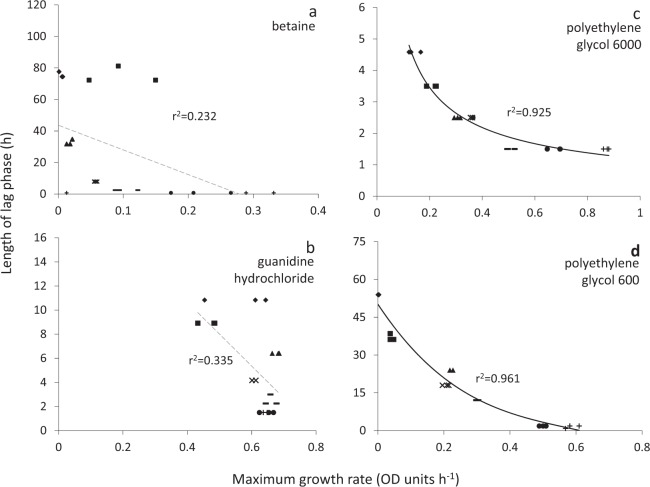
Length of lag phase versus maximum rate of growth for *Bacillus subtilis* 168 in Belitzki minimal medium broth, supplemented with a range of stressors. Media were supplemented with: (**a**) betaine at 2.56 (♦), 2.13 (■), 1.71 (▲), 1.28 (×), 0.85 (−) and 0.43 M (●), and without betaine (control; +); (**b**) guanidine hydrochloride at 150 (♦), 125 (■), 100 (▲), 75 (×), 50 (−) and 25 nM (●), and without guanidine hydrochloride (control; +); (**c**) polyethylene glycol 6000 at 50 (♦), 42 (■), 33 (▲), 25 (×), 17 (−) and 8 nM (●) and without polyethylene glycol 6000 (control; +) and (**d**) polyethylene glycol 600 at 400 (♦), 333 (■), 267 (▲), 200 (×), 133 (−) and 67 nM (●), and without polyethylene glycol 600 (control; +). Data were obtained from Fig. S7 (see *Experimental procedures)*, and trend lines showing a strong correlation between data are indicated by a solid black line (**c**,**d**), while lines fitted which indicated inconsistency between lag and growth rates (i.e. no correlation was observed) are shown as a dashed grey line (**a,b**).

**Figure 2:**
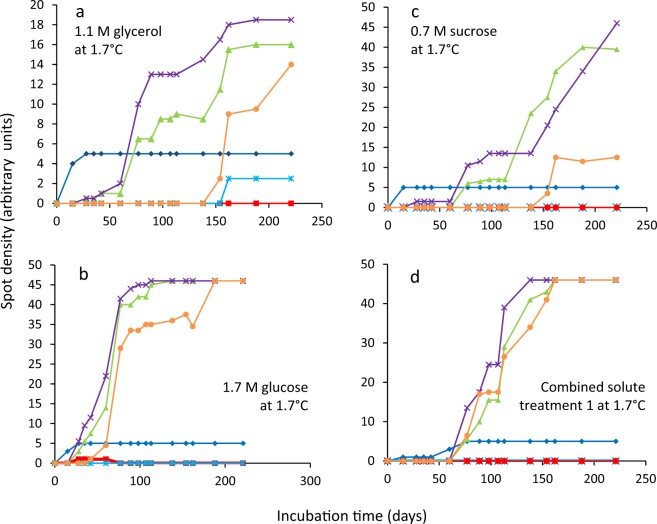
Growth curves for *Mrakia frigida* (DSM 70883) on malt-extract, yeast-extract phosphate agar (MYPiA) at 1.7 °C, supplemented with a range of stressors: (**a**) glycerol (1.1 M); (**b**) glucose (1.7 M); (**c**) sucrose (0.7 M); and (**d**) combined solute-treatment 1 (10% glycerol, 10% sucrose, 1% KCl plus 5% NaCl [w/v]). Data were obtained using Spot Test Assays (see *Experimental procedures*). For each treatment, there were six replicates; these are shown in red, blue, orange, green, purple, and turquoise on each plot.

The highest glycerol concentration at which *B. subtilis* was capable of growth (i.e. 2.71 M; Fig. S2a) corresponded to a water activity of 0.941 (Table S4). The window for growth of *B. subtilis* on glycerol-supplemented media (∼1 to 0.941) is consistent with the limits of glycerol tolerance established for *B. subtilis* and closely related strains in earlier studies^18,53,54^. *Bacillus subtilis* cultures were also grown under stresses induced by ionic osmolytes (ammonium sulphate, NaCl); non-ionic osmolytes (betaine, polyethylene glycol 600, glucose, proline, sucrose); chaotropic, osmotically-active stressors (guanidine hydrochloride, MgCl_2_); a chaotropic non-osmotic stressor (urea); and matric forces (polyethylene glycol 6000) (Figs. 1 and S2, data for NaCl- and urea-supplemented media were from Williams, 2010)^55^. For *S. cerevisiae* strains 14 and 77, the majority of cells survived throughout the dehydration period, irrespective of the subsequent rehydration treatment (Figs. S3 and S4). For *M. frigida*, growth occured at 1.7 °C in media supplemented with sucrose, glucose, glycerol, NaCl, or MgCl_2_ and combined solute-treatments: glycerol + sucrose + KCl + NaCl and glycerol + sucrose + KCl + NaCl but there was considerable variation between replicates  (Figs. 2 and S5; Table S5).

*Escherichia coli* BL21 (syn. DE3) was exposed to a range of stressors including ethanol, butanol and urea at concentrations of up to 850 mM, 120 mM, and 837 mM respectively, and the bacterium was able to grow at all concentrations tested, regardless of the stressor. Extrapolations indicate theoretical growth windows of 0 to 931 and 0 to >853 mM ethanol, and urea, respectively (Fig. S6). These upper-concentration limits are in the same concentration range as those established for *E. coli* strain BL21 (syn.  DE3) in the presence of ethanol^56^ or butanol^57^ and *E. coli* strain 31 in the presence of urea^58^.

**Figure 3:**
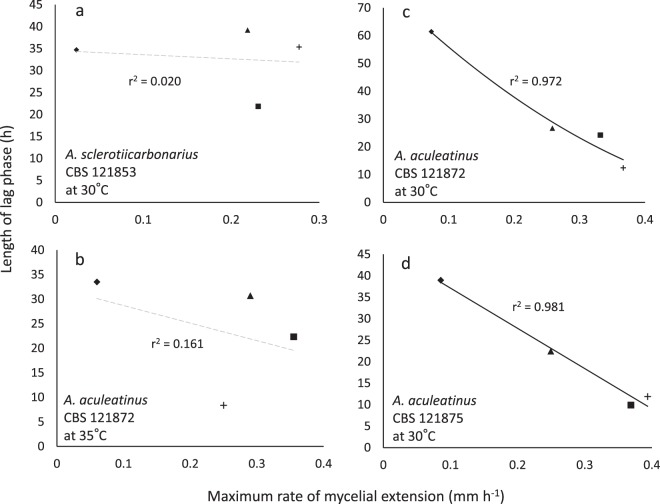
Length of lag phase versus maximum rate of mycelial extension for three *Aspergillus* strains on Robusta coffee-meal extract agar (CMEA) supplemented with glycerol: (**a**) *Aspergillus sclerotiicarbonarius* CBS 121853 at 30 °C; (**b**) *Aspergillus aculeatinus* CBS 121872 at 35 °C; (**c**) *A. aculeatinus* CBS 121872 at 30 °C; (**d**) and *A. aculeatinus* CBS 121875 at 30 °C. Media were adjusted, using glycerol (23, 9.2, 2.7 g l^−1^), to 0.900 (♦), 0.950 (▲) and 0.980 (■) water activity, respectively  (primary data were taken from Akbar and Magan^80^, see *Experimental procedures*). Control media (no glycerol added) had a water activity of 0.990 (+). In the original experiment, three replicates of each water-activity treatment were incubated at 25, 30 and 35 °C for 9 days (Akbar and Magan^80^). Trend lines showing a strong correlation between data are indicated by a solid black line (**c**,**d**), while dashed grey lines indicate inconsistency between lag and growth rates (i.e. no correlation was observed) (**a**,**b**).

During the current study, we exposed each of the model microbes to a range of stresses/stressors. This revealed considerable variation in the relationship (or lack of relationship) between lag and growth rates for each individual microbe, depending on stressor. It also revealed that the same stressors do not necessarily give the same result for different microbes. For instance, r^2^ values for aspergilli and *Bacillus subtilis* on glycerol-supplemented media ranged from 0.020 to 0.972 (Figs. 3 and S2a). Whereas there have been earlier studies in which an individual microbial strain has been exposed to a number of mechanistically distinct solute-induced stresses^59–62^, tolerance to six or more individual solute stressors has only been studied for a relatively small number of microbial strains. For fungi, these belong to the following species: the extreme xerophile *Aspergillus wentii*^47^; mesophilic ascomycetes *Aspergillus oryzae*^63^; *Saccharomyces cerevisiae*^32,64^; fission yeasts *Schizosaccharomyces japonicas*, *Schizosaccharomyces malidevorans*, *Schizosaccharomyces octosporus*, *Schizosaccharo-myces pombe*, and *Schizosaccharomyces slooffiae*^65^; and the entomopathogens *Beauveria bassiana, Metarhizium anisopliae*, and *Paecilomyces farinosus*^66–68^. For bacteria: *Bacillus subtilis* (current study); the solvent-tolerant *Pseudomonas putida*^32,69–71^; the environmentally tenacious *Mycobacterium parascrofulaceum* and *Mycobacterium smegmatis*^72^; and psychrophiles: *Glaciecola punicea, Psychrobacter glacincola, Psychrobacter urativorans*, and *Sporosarcina psychrophilia*^73^; the xerotolerant and acid-tolerant bacterium *Gluconacetobacter diazotrophicus*^74^ and the halotolerant *Ochrobactrum* sp.^75^. The bacteria that were used in other studies are generally robust, stress-tolerant species, yet *B. subtilis* (168) exhibited comparable solute-tolerances to many of them. For instance, *Glaciecola punicea, Psychrobacter glacincola, Psychrobacter urativorans*, and the mycobacteria could grow up to ≥1000 mM MgCl_2_, and the limit for *B. subtilis* is >1710 mM (Fig. S2d). Similarly, the most-NaCl tolerant of the bacteria studied previously (*M. parascrofulaceum* and *M. smegmatis*) which can grow up to ≥1710 mM NaCl, was only able to tolerate 50% more NaCl than *B. subtilis* 168 (upper limit = 1140 mM^55^). *Pseudomonas putida* and the mycobacteria (*M. parascrofulaceum* and *M. smegmatis*) grow up to 2120 and 4792 mM glycerol, respectively, and the tolerance limit for *B. subtilis* is >2710 mM (Fig. S2a), and all these bacteria have a comparable upper limit for urea; i.e. 950, 832, and 755 mM, respectively^32,55,72^. The water-activity values at which the xerophiles under study can germinate are considerably lower than the values at which mycelial growth or spore germination ceases (i.e. in the range 0.880–0.920) for non-xerophilic species such as *Beauveria bassiana, Metarhizium anisopliae, Paecilomyces farinosus*, *S. cerevisiae* and *A. oryzae*^63,76–78^.

### Lag phase can sometimes vary independently of maximum rates of germination/growth

**Figure 4:**
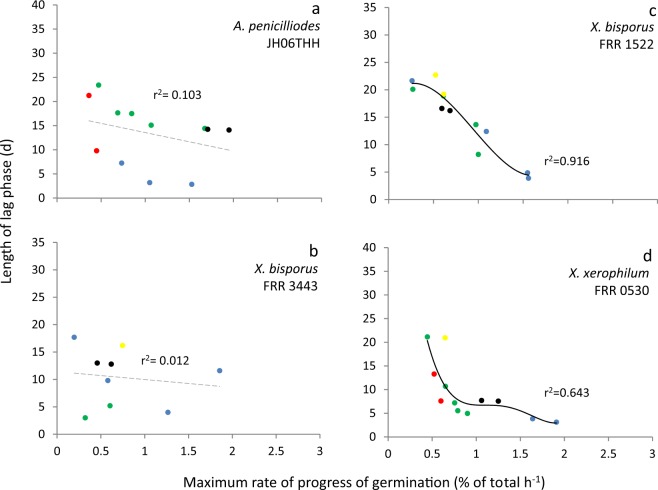
Length of lag phase versus maximum rate of germination for four strains of extremophilic fungi: (**a**) *Aspergillus penicillioides* JH06THH; (**b**) *X. bisporus* FRR 3443; (**c**) *X. bisporus* FRR 1522; and (**d**) *Xerochrysium xerophilum* FRR 0530. Length of the pre-germination phase and maximum rate of germination were determined on malt-extract  yeast-extract phosphate  agar (MYPiA) supplemented with diverse stressor(s) and incubated at 30 °C for up to 50 days. Media were supplemented with: glycerol (red dots), at 7.0 and 7.1 M, with water-activity values of 0.707 and 0.664, respectively; glycerol (5.5 M) + NaCl at 0.5, 1.0, 1.5, 1.6 and 1.7 M (green dots), with water-activity values of 0.765, 0.741, 0.709, 0.692 and 0.668, respectively; glycerol (5.5 M) + sucrose at 0.25, 0.50, 0.65 and 0.80 M (blue dots), with water-activity values of 0.734, 0.699, 0.674 and 0.637, respectively; glycerol (5.5 M) + glucose at 0.8 and 1.0 M + fructose at 0.80 and 1.0 M (yellow dots), with water activities of 0.694 and 0.649, respectively; and glycerol (5.5 M) + NaCl (0.5 M) + sucrose at 0.3 and 0.5 M (black dots), with water-activity values of 0.701 and 0.685, respectively. Data were obtained from Fig. S1 (see *Experimental procedures)*, and trend lines showing a correlation between data (whether strong or moderate, see *Experimental procedures)* are indicated by a solid black line (**c**,**d**) while lines fitted which indicated inconsistency between lag and germination rates (i.e. no correlation was observed) are shown as a dashed grey line (**a**,**b**).

**Figure 5:**
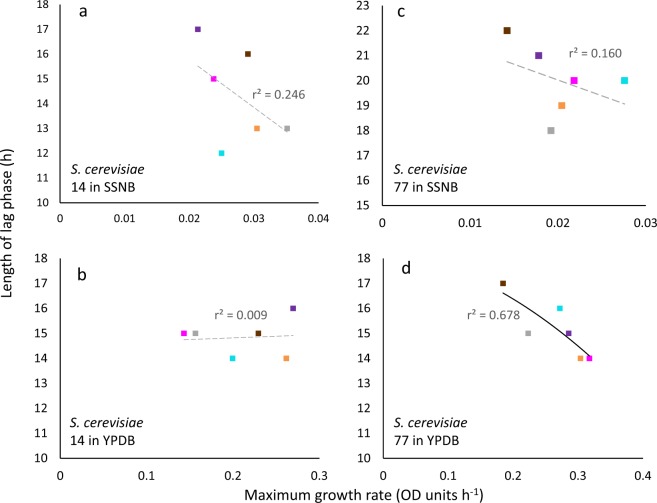
Length of lag phase versus maximum rate of growth at 23 °C: strain 14 in salt-supplemented nutrient broth (SSNB) (**a**) and yeast extract peptone dextrose broth (YPDB) (**b**); and strain 77 in SSNB (**c**) and YPDB (**d**). Cells suspended in water (not subjectied to any dehydration treatment) (pink squares), and cells suspended in 1 M xylitol (no dehydration treatment) (orange squares), were used as controls. For dehydrated cells, rehydration treatments were either rapid, using water (grey squares); rapid, using 1 M xylitol (brown dots); gradual, using water vapour then rapid in water (purple squares); or gradual rehydration with water vapor, then rapid in 1 M xylitol (turquoise squares). For strong correlations, trend lines are indicated by a solid black line (**d**), while inconsistency between lag and growth rates (i.e. no correlation was observed) is indicated by a dashed grey line (**a**–**c**).

**Figure 6:**
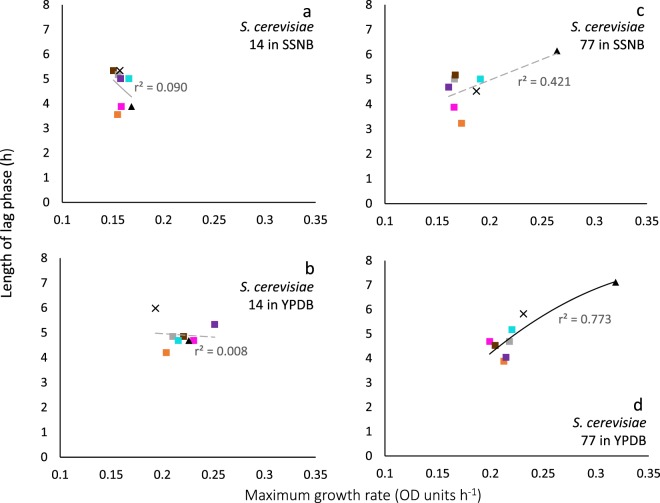
Length of lag phase versus maximum rate of growth for *Saccharomyces cerevisiae* at 30 °C: strain 14 in salt-supplemented nutrient broth (SSNB) (**a**) and yeast extract peptone dextrose broth (YPDB) (**b**); and strain 77 in SSNB (**c**) and YPDB (**d**). Cells suspended in water (not subjected to any dehydration treatment) (pink squares), and cells suspended in 1 M xylitol (no dehydration treatment) (orange squares), were used as controls. For dehydrated cells, rehydration treatments were either rapid, using water (grey squares); rapid, using 1 M xylitol (brown squares); gradual, using water vapour then rapid in water (purple squares); or gradual rehydration with water vapor, then rapid in 1 M xylitol (turquoise squares) or for xylitol dehydrated cells; gradual, using water vapour then rapid in water (×); and gradual, using water vapour then rapid in 1 M xylitol (▲). Trend lines showing a strong correlation between data are indicated by a solid black line (**d**), while inconsistency between lag and growth rates (i.e. no correlation was observed) is indicated by a dashed grey line (**a**–**c**).

**Figure 7:**
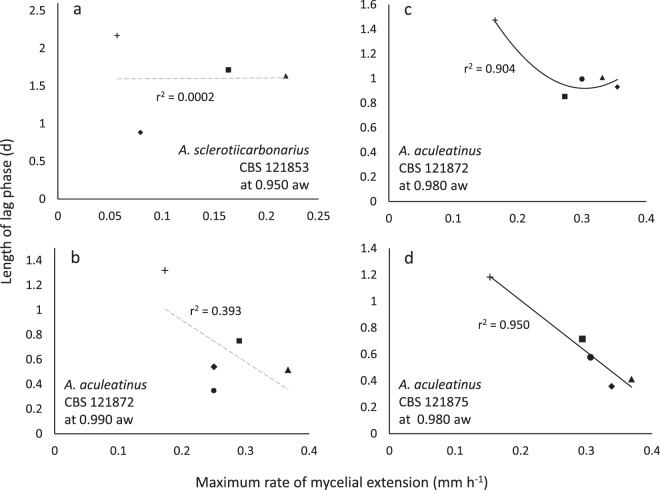
Length of lag phase versus maximum rate of mycelial extension for three *Aspergillus* strains on Robusta coffee-meal extract agar (CMEA) supplemented with glycerol: (**a**) *Aspergillus sclerotiicarbonarius* CBS 121853 at a water activity  (aw) of 0.950; (**b**) *Aspergillus aculeatinus* CBS 121872 at a water activity of 0.990; (**c**) *A. aculeatinus* CBS 121872 at a water activity of 0.980; (**d**) and *A. aculeatinus* CBS 121875 at a water activity of 0.980 (primary data were taken from Akbar and Magan^80^, see *Experimental procedures*). Three replicates of each treatment were incubated at 20 (+), 25 (■), 30 (▲), 35 (♦), and 37 °C (●) for 9 days. Trend lines showing a strong correlation are indicated by a solid black line (**c**,**d**), while dashed grey lines indicate inconsistency between lag and maximum rate of mycelial extension (i.e. no correlation was observed) (**a**,**b**).

There was no correlation between lag phase and maximum rates of germination/growth for some of the fungal germination- and bacterial growth datasets. In general, length of lag phase increased progressively at lower water-activities for *X. bisporus* FRR 3443 (Fig. S1b), but maximum rates of germination did not decline progressively regardless of type of stressor/stressor combination (Figs. 4b and S1b; maximum gradients of the linear trend lines are shown in Table S6). Similarly, there was inconsistency between length of lag and the maximum growth rates for the *B. subtilis* 168 cultures in guanidine hydrochloride- and betaine-supplemented media (Figs. 2a,b and S7a,b). For these xerophile strains and *B. subtilis* cultures, plots of lag-phase versus germination/growth rates were scattered, and/or the line of best fit was approximately horizontal. The r^2^ value for plots of *B. subtilis* growth in betaine- and guanidine hydrochloride-supplemented media were 0.232 and 0.335, respectively (Fig. 1a,b), and for *X. bisporus* (FRR 3443) and *A. penicillioides* JH06THH, r^2^ values were only 0.012 and 0.103, respectively (Fig. 4a,b; Table S6). Other xerophiles for which the lag phase and germination rates did not correlate were *X. bisporus* FRR 0025 and *E. echinulatum* FRR 5040 (r^2^ = 0.208 and 0.298, respectively; Fig. S8g,d). The psychrophilic yeast *M. frigida* DSM 70883 also revealed a disconnect between lag-phase and growth rates for 10% w/v glycerol- and 30% w/v glucose-supplemented media (r^2^ = 0.144 and 0.415, respectively; Fig. S9c,d). For diverse microbes, attempts to fit trend lines to a number of plots (for diverse microbes) yielded lines that are close to horizontal (Figs. 3a, 4b, 5b, 6b and 7a). For instance, the gradients for trend lines of *A. sclerotiicarbonarius* in glycerol-supplemented media over a range of temperature (Fig. 3a) and water activity (Fig. 7a) were −0.395 and 0.0907, respectively (Table S6). Similarly, for *S. cerevisiae* that had been dehydrated and rehydrated rapidly in sterile distilled water or with a 1 M xylitol solution at 23 °C, the gradient of the trend line for lag and maximum growth rates was −1.31 (Fig. 5b; Table S6). A horizontal trend line shows that length of lag phase does not change even though maximum rate of germination varies. This inconsistency indicates that lag phase cannot be used to predict germination rates.

**Figure 8:**
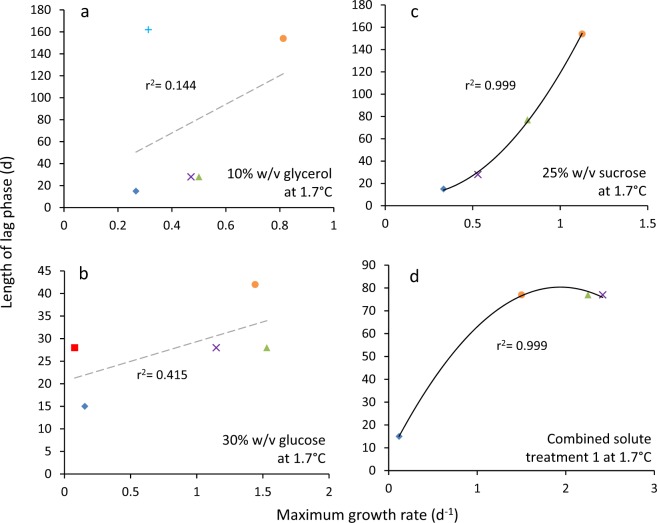
Length of lag phase versus maximum rate of growth *Mrakia frigida* (DSM 70883) on malt-extract, yeast-extract phosphate agar (MYPiA) at 1.7 °C, supplemented with a range of stressors: (**a**) glycerol (1.1 M); (**b**) glucose (1.7 M); (**c**) sucrose (0.7 M); and (**d**) combined solute-treatment 1 (10% glycerol, 10% sucrose, 1% KCl plus 5% NaCl [w/v]). For each treatment, there were six replicates; these are shown in red, blue, orange, green, purple, and turquoise on each plot. Data were obtained from Fig. 2; see *Experimental procedures*. Trend lines showing a strong correlation between data are solid and black (**c**,**d**), while dashed grey lines indicate inconsistency between lag and growth rates (i.e. no correlation was observed) (**a**,**b**).

In some of the cases where there was no correlation between length of lag phase and subsequent rates of germination/growth, there is nevertheless evidence that cultures experienced considerable stress during the lag phase. For instance, the culture conditions under which lag phase and growth rates were determined for *B. subtilis* and *A. penicilliodes* were more stressful than those of the preculture conditions (because the former included the presence of solute stressors in the medium) (Table S2). Accordingly, growth rates were reduced at high concentrations of stressors (Figs. 1a,b, 4a,b, S1a,b, S7a,b and S8d,g). *Aspergillus. aculeatinus* was subcultured at optimal temperature, however the temperatures under which lag phase and growth rates were determined were higher than optimal, adding to the considerable stress during the lag phase. The disconnects between the lag phase and growth rate that are apparent in Figs. 1a,b, 3a, 4a,b, 5a–c, 6a–c, 7a,b, 8a,b, S1b, S3a,b, S7a,b, S8d,g and S9c,d are consistent with the findings of some other studies. For instance, Ding *et al*.^79^, characterized lag-phase adaptation in mutants of *S. cerevisiae* which over-expressed genes implicated in acid-stress tolerance. Over-expression of genes which code for a protein thought to increase vacuolar proton-pumping ATPase activity, PEP3, also shortened the lag phase. However, over-expression of PEP3 decreased length of lag phase but did not alter the rate of subsequent growth. In a study of two *Aspergillus* species, food-spoilage strains were cultivated over a range of water-activity values, in glycerol-supplemented nutrient media, and incubated over a range of temperatures^80^. We analysed the relationship between lag phase and maximum rate of mycelial extension of these fungi and found that, in some cases there was inconsistency between these parameters; for *A. sclerotiicarbonarius* CBS 12183 at 0.950 water activity and *A. aculeatinus* CBS 121872 at 0.990 water activity, r^2^ = 0.0002 and 0.393 respectively (Fig. 7a,b). For *A. aculeatinus* CBS 121872, there was a decrease of growth rate at high temperatures (a phenomenon often seen at supra-optimal water activity^49,66^) which contributed to the scatter. Furthermore, when plots were constructed for the same fungal strains in relation to each individual temperature, data for *A. sclerotiicarbonarius* CBS 121853 at 30 °C, and *A. aculeatinus* CBS 121872 at 35 °C were also scattered (Fig. 3a,b respectively).

**Figure 9:**
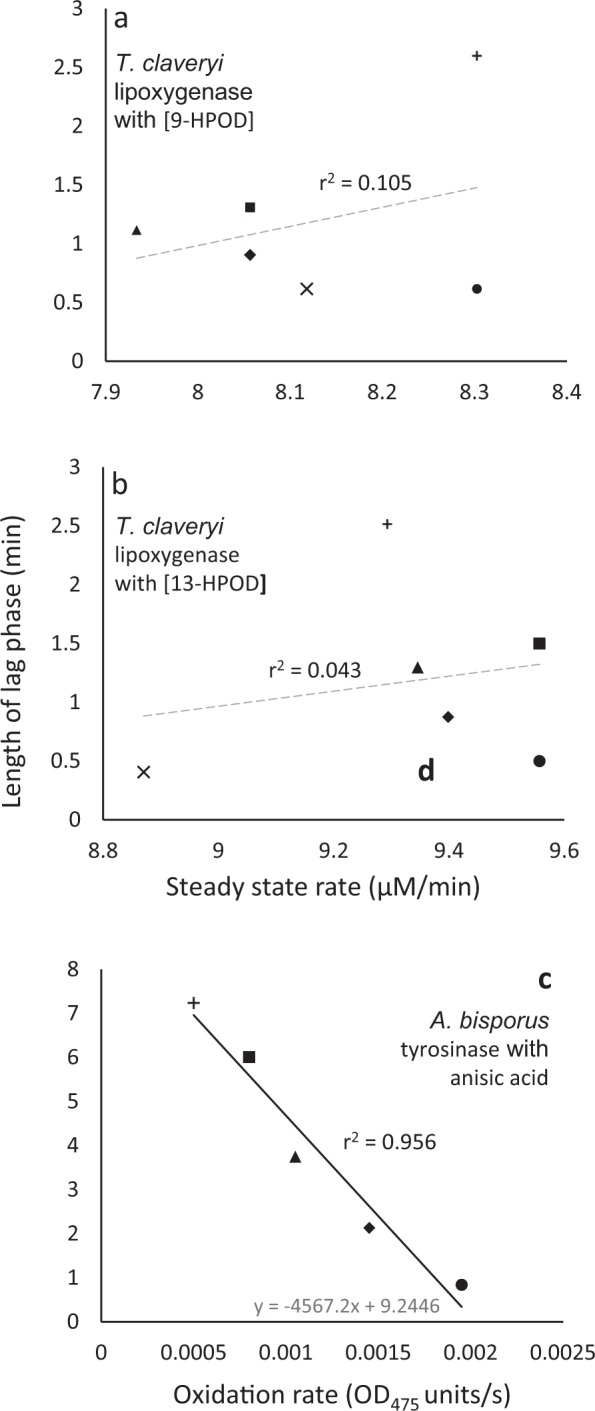
Length of lag phase versus steady-state rate for *T. claveryi* lipoxygenase activity exposed to two hydroperoxy octadecadienoic acids; (**a**) 9-HPOD and (**b**) 13-HPOD; and *A. bisporus* tyrosinase exposed to a carboxylic acid; (**c**) anisic acid. For the hydroperoxy octadecadienoic acids, media composition consisted of 180 µM linoleic acid, and 0.01 unit of *Terfezia claveryi* LOX in sodium borate buffer at pH 10.0 and media were supplemented with: (**a**) 9-HPOD at 16.28 (×), 14.99 (●), 7.54 (♦), 5.23 (▲), and 2.66 mM (■) and without 9-HPOD (control; +); (**b**) 13-HPOD at 19.54 (×), 0.81 (●), 0.32 (♦), 0.24 (▲), and 0.16 mM (■) and without 13-HPOD (control; +). The reaction medum (3 ml) for the carboxylic acid consisted of 0.5 mM of tyrosine in 50 mM sodium phosphate buffer (pH 6.8), mushroom tyrosinase 33.3 µg/ml and media were supplemented with (**c**) anisic acid at 3 (●), 2 (■), 1 (▲), and 0.4 (♦) mM, and without anisic acid (control; +). Trend lines showing a strong correlation between data are solid and black (**c**,**d**), while dashed grey lines indicate inconsistency between lag and steady-state rate (i.e. no correlation was observed) (**a**,**b**).

A study of a food-borne, pathogenic bacterium, *Chronobacter*, revealed that lag phase increased after acid stress, but lag was not proportional to subsequent growth rate^26^. Similarly, in relation to enzyme systems, studies of *T. claveryi* lipoxygenase activity during exposure 13- and 9-hydroperoxide derivatives (HPOD) of linoleic acid (i.e., 13-HPOD and 9-HPOD respectively) indicated that lag phase was not proportional to tolerance of oxidative stress. Data for rates of catalytic activity during steady state, when plotted against lag phase, were scattered, with r^2^ values of 0.105 for 9-HPOD and 0.043 for 13-HPOD (Fig. 9a,b). For a microbial population that has been unable to grow due to unfavourable environmental conditions, cells will sense changes in the intra- and/or extracellular milieu that facilitate growth, thus triggering the lag phase. Whereas there is a substantial literature about the end of lag and transition to exponential growth rate, there is little information about how (in terms of cellular biology) we define the start of lag. According to Penfold^4^, “By bacterial lag, we understand, the interval between the inoculation of a bacterial culture and the time of commencement of its maximum rate of growth.” It may be more appropriate to define lag from the time when genetic and metabolic adaptations begin. When microbial cells are placed into a fresh culture medium, changes in gene expression have been detected within 4 minutes, for 1119 genes^12^. The question remains, both in conceptual and practical terms, how to determine that lag has begun? Do we assume this occurred at time 0^81,82^ or at some point between 0 and 4 mins? Regardless of this, and as detailed by Pirt^83^, “the requisite conditions for growth of biomass in culture are:

- a viable inoculum,
- an energy source,
- nutrients to provide the essential materials from which biomass is synthesized (it may be that metabolites leaking from the cell can at as nutrients to facilitate entry into lag proper),
- absence of inhibitors which prevent growth, [and]
- suitable physicochemical conditions.”

Each of these conditions is characterized by complexity and so represents a black box when considering implications for microbial cell biology. However, it is well-established that many phenotypic, physiological and molecular changes occur during lag phase that are not stress responses, and the environmental changes that can induce lag phase do not necessarily induce stress. Genes or enzymes that are generally expressed or required to keep cellular systems functional are said to be involved in housekeeping^84,85^; these can include genes/proteins involved in DNA replication/repair, glycolysis, and construction of the cytoskeleton. Levels of mRNA for housekeeping genes are not always constant because housekeeping activities can vary in intensity depending on environmental and other factors. Furthermore, some housekeeping proteins are stable, so the corresponding genes are expressed at low levels. This said, there are housekeeping activities that are imperative to the lag phase of microbes. Accordingly, concentrations of DNA repair proteins (e.g. endonucleases) and DNA precursers increase during lag, thus facilitating the repair of any damaged DNA prior to chromosome replication^8,12^. The (picomolar) concentrations of metabolic intermediates in central carbon metabolism typically increase in the cytosol during the lag phase. Some of these facilitate increased glycolytic flux helping to fuel increased protein synthesis^8,12^. In turn, increases in cell-available energy, and protein synthesis, enable increased lipopolysaccharide production needed for cell-membrane repair and synthesis, and cellular division^12,86^.

### Growth rates can sometimes vary with minimal variations in lag phase

Under some stress regimes, microbial growth rates varied considerably whereas lag phase was relatively consistent. For example, *B. subtilis* exhibited growth rates between 0.0068 and 0.651 OD units h^−1^ when grown in liquid media supplemented with ammonium sulphate (over a range of concentrations; Figs. 1c and S7c). In each case, lag phase was relatively short (between 1.5 and 7.5 hours), so whereas there was a 96-fold difference in growth rates, the lag phases of these cultures only showed a five-fold variation (Figs. 1c and S7c). The same phenomenon was seen for *S. cerevisiae* strains that had been exposed to different dehydration-rehydration treatments and then inoculated into a liquid medium. For instance, *S. cerevisiae* 14 grown at 23 °C YPDB (Figs. 5b and S3b) exhibited growth rates that ranged from 0.14 to 0.27. The maximum rate was 100% greater than the minimum, whereas lag phases varied from only 14 to 16 hours. For mycelial extension of *A. sclerotiicarbonarius* when grown over a range of water-activity values (at 30 °C; Fig. 3a), growth rates varied by 11.5-fold whereas lag phase varied by only two-fold. As discussed above (in *Lag phase can sometimes vary independently of maximum rates of germination*/*growth*), it might be expected that the length of lag changes when environmental conditions and/or the degree of stress changes. Indeed, it seems counterintuitive that very different levels of stress – as evidenced by a wide variation in growth rates – can occur with a small or negligible change in lag phase when the inoculum came from a single source (Figs. 1c, 3a, 5b, 6b and S7c). These data imply that, despite the considerable difference in the intensity of stress, there was a minimal or very rapid change required to adapt to the conditions imposed. This phenomenon was observed in *E. coli*, and was mostpronounced under urea-induced stress where the variation in growth rates was an order of magnitude greater than variation in lag. Whereas maximum growth rate decreased by 8.4-fold as urea concentration increased (between 600 to 900 mM), the corresponding extension of lag phase was only 1.7-fold (Fig. S10c). In some of the cases where growth rates varied considerably but lag phase was relatively consistent, cultures may have experienced considerable stress during the lag phase (Figs. 1c, 3a and S7c), but not in others (5b; 6b; S3b and S4b). For instance, the culture conditions for determinations of lag phase and growth rates for *A. sclerotiicarbonarius* were more stressful than preculture conditions; due to solute stressors and supra-optimal temperatures (Table S2).

### Lag phase can sometimes correlate with the maximum rates of germination or growth

Generally, the microbes under study are more tolerant to solute-induced stresses than other filamentous fungi (for the xerophiles), yeasts (for *S. cerevisiae*) and mesophilic bacteria (especially *B. subtilis*)^16–18,48^. In relation to the aim of our study, however, the question remains whether lag phase is ever indicative of stress.

We found strong correlations between length of lag and subsequent rates of exponential growth under some conditions; for *B. subtilis*, germination for some fungal xerophiles, and for colony formation of *M. frigida* (Figs. 1c,d, 4c,d, 8c,d and S2b–f; Table S6). In the cases where strong correlations were observed (r^2^ > 0.810), the gradient (m) varied between −3.878 and −4567 (Table S6). For instance, the fungal xerophile *X. bisporus* (FRR 1522) was progressively more stressed on media with lower water-activity; lengths of lag phase were longer and gradients of exponential-phase growth curves less steep on media with high concentrations of glycerol (Fig. S1c). The length of lag phase was strongly correlated with subsequent rates of germination for *X. bisporus* (FRR 1522) (r^2^ = 0.916; Figs. 4c and S1c; Table S6). In addition, there was a number of other plots of lag versus germination rate where r^2^ ranged from 0.639 to 0.714, indicating moderately good correlations (Figs. 4 and S8). These included *X. xerophilium* FRR 0530, *X. bisporous* FRR 2347, *E. repens* JH06JPD and *E. amstelodami* FRR 2792 (r^2^ = 0.643, 0.639, 0.666 and 0.714; Figs. 4d and S8c,f,h; Table S6). Whereas lag phase of *S. cerevisiae* 14 correlated weakly with exponential growth rate (Figs. 5a,b and 6a,b), lag phase and growth rate showed a moderately good correlation for S. cerevisiae 77 on YPDB at 23 °C and on SSNB at 30 °C (r^2^ = 0.678 and 0.773, respectively ; Figs. 5d and 6d). The lag phase of *B. subtilis* strongly correlated with exponential growth rate in media supplemented with ammonium sulphate, polyethylene glycol 600, proline, MgCl_2_, sucrose and polyethylene glycol 6000 (r^2^ = 0.880, 0.961, 0.941, 0.901, 0.876 and 0.973, respectively; Figs. 1c,d and S11b,d–f). In addition, there was a moderately good correlation for the *B. subtilis* in media supplemented with glycerol; r^2^ = 0.593 (Fig. S2a). The lag phase of *M. frigida* was strongly correlated with subsequent rates of growth on media supplemented with sucrose (0.7 M), sucrose (1.5 M), glycerol (2.7 M), NaCl (2.5 M), and a combination of solutes (i.e., 0.55 M glycerol, 0.3 M sucrose, 0.1 M KCl plus 2.5 M NaCl) (r^2^ = 0.999, 0.956, 0.947, 0.842 and 0.999 respectively; Figs. 8c,d and S9a,d,e). In addition, there were moderately good correlations for *M. frigida* on media supplemented with MgCl_2_ (1 M), glycerol (4.3 M), glucose (2.8 M), and a combination of solutes (1.1 M glycerol, 0.3 M sucrose, 0.1 M KCl plus 2.5 M NaCl) (r^2^ = 0.771, 0.703, 0.687 and 0.743 respectively; Fig. S9b,c,f,g).

Correlations between lag and growth rates can also be found in the datasets we analysed from other researchers. For example, Li and Torres^87^ carried out a study of Gram-negative and Gram-positive bacteria (of the phyla Proteobacteria and Firmicutes) in liquid media supplemented with the mechanistically diverse solute-stressors NaCl, glycerol, and sucrose^47^. Analyses of length of lag versus exponential growth rate from 15 cultures/treatments^87^ revealed correlations between these two paremeters (13 strong correlations and two moderate correlations), regardless of bacterium or stressor (Fig. S12). For instance, the Gram-negative bacteria *Pseudomonas fluorescens* and *Salmonella typhimurium*, exhibited strong correlations between lag phase and exponential growth rate in media supplemented with NaCl, glycerol, and sucrose (r^2^ = 0.893; 0.921; 0.875, respectively for *P. fluorescens* [Fig. S12a,f,k] and 0.895; 0.963 and 0.892, respectively for *S. typhimurium* [Fig. S12d,I,n]). There were also strong correlations for the Gram-positive *Brochothrix thermosphacta* when media was supplemented with NaCl and glycerol (r^2^ = 0.940 and 0.915, respectively; Fig. S12b,g), and moderately a good correlation for media supplemented with sucrose (r^2^ = 0.723; Fig. S12l). For the Gram-positive bacterium *Enterococcus faecalis*, lag phase and exponential growth rate correlated strongly in media supplemented with NaCl (r^2^ = 0.842; Fig. S12c), and correlated moderately for media supplemented with glycerol or sucrose (r^2^ = 0.607 and 0.762, respectively; Fig. S12h,m). For *S. aureus,* the lag phase correlated strongly with exponential growth rate in media supplemented with NaCl, glycerol, and sucrose (r^2^ = 0.833, 0.913 and 0.857, respectively; Fig. S12e,j,o).

Where there were strong correlations between lag phase and maximum rates of subsequent germination/growth, there is evidence that cultures experienced considerable stress during the lag phase. For instance, determinations of length of  lag and growth rates for *Pseudomonas fluorescens* and *Salmonella typhimurium* were carried out under more-stressful conditions (due to the solutes present) than those of the precultures (no stressors present) (Fig. S12a,d,f,I,k,n; Table S2). That lag phase can sometimes indicate the level of stress is also consistent with the data reported by others. For instance, studies of oxidative stress in *S. cerevisiae* BY4741 grown in batch culture found that length of lag phase is in approximate agreement with rates of exponential growth 1^20^. In the *Aspergillus* study by Akbar and Magan^80^, there was a strong correlation between length of lag phase and maximum growth rate for some strains, including *A. aculeatinus* CBS 121872 at 0.980 water activity (r^2^ = 0.904) and *A. aculeatinus* CBS 121875 at 0.980 water activity (r^2^ = 0.950) (Fig. 7c,d). Furthermore, when growth curves of these strains were obtained for cultures on glycerol-supplemented media (over a range of water activities), there were also strong correlations between lag phase and maximum growth rates, depending on strain and temperature (Fig. 7c,d). Similarly, functional analyses of mutant strains have shown that lag phase is proportional to glycolaldehyde-induced inhibition, in *S. cerevisiae* BY4743^43^, and osmotic stress, in *Salmonella enterica*^88^. A correlation between lag phase and stressor concentration was also observed in *Bacillus* strain TCL when exposed to oxidative stress induced by chromium^36^. A study of *Listeria innocua* exposed to stresses caused by UV-C and oxidative damage found that lag increased 13-fold whereas the growth rate was reduced by only 45%; nevertheless, the former still correlated with the latter^89^.

Many changes and adaptations which must take place during the microbial lag phase (whether for vegetative growth, germination, or other processes) relate directly to cellular stress and response and adaptation to stress. For instance, desiccated spores that imbibe water (a process that can be, in part at least, driven by ATP^90,91^) experience rehydration-related and dilution stresses (which may include hypo-osmotic shock); or new environmental conditions may necessitate the production of protein-stabilisation proteins, compatible solutes, or systems to remove reactive oxygen species^92–94^. It is therefore no surprise that lag can correlate with subsequent rates of growth or (presumably) with other indicators of cellular stress. Conversely, length of lag may not be determined by intensity of stress and, accordingly, lag may correlate weakly with growth rate, or not at all (see above).

The activities of housekeeping genes or protiens may increase (or decrease) under stress and vice versa. For instance, cellular changes in response to mild- to moderate levels of stress typically include increases in protein synthesis and upregulation of energy generation^62^. These, in turn, may require increased flux through glycolysis and/or upregulation of other so-called housekeeping functions. Stress-specific responses can also be upregulated during lag phase; these may relate to rehydration-induced stresses (when propagules imbibe water for instance) or other stressful events/stress parameters.

Other stress-induced changes that occur during lag phase include: protein-stabilisation proteins which mitigate against the chaotropicity of ethanol in *E. coli*^94^; increased expression of proline- and glycine-betaine transporters in *Bacillus cereus* under NaCl (osmotic) stress^91^; trehalose accumulation in response to a heat ramp from 28 to 41 °C in *Saccharomyces cervisiae*^93^; and acid-shock protein production in *Salmonella typhimurium* at low pH^95^.

It would therefore be expected that length of lag can sometimes act as a useful indicator of stress, and that at other times it may do so only weakly or not at all. Whereas both the lag- and stationary phases of batch cultures are characterized by very slow (or even zero) growth rates, it could be argued that the lag phase differs from the both the stationary- and late exponential phases in this way. Exponential growth can be seen as an expression of completely functional cell division processes, though these rarely, if ever, attain their theoretical optima. A variety of things can impede or prevent cell division; oxidative stress, lack of a key nutrient, cell division inhibitors, environmental stress signals, etc. So, observations of a lack of correlation between lag phase/exponential growth rate ratios and microbial responses to stress might be expected. When viewed this way, microbial lag phases seem analogous to cell senescence of animal and plant cells…cell senescence is generally viewed to occur with blockage of eukaryotic cell cycle check points [S, G2, M, G1] while metabolism continues. Markers for cell scenescence are known - e.g. p16, p21, p53, etc. - and nearly all are inhibitors of specific cell cycle check point targets [e.g. kinase inhibitors or activators] or upstream effectors of these inhibitors. We beleive that analogous processes are going on during microbial lag phases. Consistent with this, although there were strong correlations between lag phase and rates of exponential growth/germination in some cases and no correlation for others, the r^2^ values for Figs. 1–7, S2, S8–S10 and S12 were more or less proportional to the steepness of gradients (see also Table S6). There was a total of 16 datasets exhibiting a moderately good correlation (see above), and two plots which indicate a weak correlation. The latter were *A. penicillioides* JH06THJ on glycerol+NaCl−; glycerol+sucrose− and glycerol+NaCl+ sucrose-supplemented media and *E. coli* BL21 (syn. DE3) on Luria-Bertani broth supplemented with ethanol (r^2^ = 0.512 and 0.544 respectively; Figs. S8b and S10a; Table S6).

## Concluding remarks

Definitions and studies of lag phase have almost exclusively related to microbes in batch cultures. Lag, however, occurs in virtually all types of microbial habitat within the biosphere, including times when populations come out of a prolonged stationary phase. A lag phase may also occur as a deviation from exponential growth. Furthermore, lag takes many forms and is characterised by complexity in relation to heterogeneity within populations of individual microbes, and in the context of evolutionary biology. For instance, a cell’s environmental and life history can determine rates (and other aspects) of adaption including length of lag^96^. Many studies also show phenotypic and behavioural heterogenity within populations of individual strains^97–100^. For instance, a study of *S. cerevisiae* cells from a glucose-containing (sole carbon source) medium were placed into a maltose-containing medium revealed profound differences in lag phase times. Indeed, up to 90% of cells never left the lag phase during the experimental period^101^, which may indicate genetic and/or epigenetic variation within the population; either of which can provide the basis for evolutionary changes^99^. Further examples can be found in the recent review by Bertrand^86^. In some cases, microbial cells can remain dormant for an indefinite period (independently of environmental conditions) even though they are viable^102,103^. This can sometimes be mistaken for lag. So, whereas much is known about lag phase, some aspects of lag also remain unpredictable and enigmatic. It should also be noted that the lag phase can involve the up-regulation as well as down-regulation of stress responses^104^. In relation to lag as a measure of vitality or stress tolerance, we believe that measures of lag (or single-point determinations of biomass) represent a precarious means to assess cellular stress.

In the current study, we analysed phylogenetically and ecophysiologically diverse microbes under various types of solute- and water-induced stress. Given that life is based on water, these stresses are omnipresent and potent. Indeed, they act as a paradigm for evolution and adaptation, the environmental challenges that life is continutously exposed to, and the functionality and survival microbial cells and their communities^13,105^. The study revealed that lag phase can be proportional to subsequent rates of growth/germination and so, in these cases at least, might be considered an indicator of cellular stress. However, we also found that length of lag is frequently unrelated to rates of growth/germination. For each type of microbe and each type of stress, length of lag phase can both correlate with, and be independent of, stress. Lag phase can vary independently of exponential growth rate and, in other cases, growth rates can vary when lag phase is constant. Regardless of the microbial system or microbial process, therefore, length of lag phase can correlate with and can be inconsistent with growth rate. Both these trends were observed whether stress treatments are differed quantitatively (Figs. 1 and S2) or qualitatively (Figs. 5 and 6); for fungi as well as bacteria (Figs. 1, 3–7, S2, S8–S10 and S12); in relation to diverse environmental parameters and cellular-stress treatments (water activity, chaotropicity, temperature, dehydration-rehydration); whether cultures were planktonic (Figs. 4, 5 and 7) or germinating or growing on a solid surface (Figs. 3, 4 and 7); and even for enzyme systems (Fig. 9). When the same *B. subtilis* strain is subjected to solute-induced stresses, lag phase can be strongly proportional to, independent of, or weakly correlated with the level of stress; depending on the solute stressor, but regardless of whether the latter is ionic or non-ionic (Figs. S7 and S11). Similarly, when *A. aculeatinus* CBS 121872 is cultured at different water activities, the lag phase may be inconsistent with or strongly correlated with exponential growth rate, depending on water activity (Fig. 7). Furthermore, when the same strain was incubated at a range of temperatures, both scatter plots and strong correlations were obtained (Fig. 3). It may frequently be difficult, if not impossible, to predict which cultures and/or stressors/stress regimes and/or stress parameters/mechanisms will be produce a lag phase that is primarily determined by stress, and which will not. In conclusion, therefore, single-point determinations of growth, should not be assumed to be indicative of microbial stress. In other words, lag phase is not a reliable measure of microbial stress. Whereas exponential growth rate is not ideal to define or quantify cellular stress^13^, it may actually be the best proxy we currently have.

The findings of this study give rise to a number of unanswered questions in addition to those discussed above:

1. Apart from cellular growth, a lag phase can occur at various levels of complexity within biology; the macromolecular system^106,107^, the organism^108,109^, the ecosystem^110,111^, and evolutionary biology^112,113^. Furthermore, the biophysical events and chemical reactions which can drive biological processes may have their own lag phases; e.g. in relation to phase shifts in oscillatory processes (such cytoskeletal force generation and circadian clocks) or various types of friction (e.g. because of diffusion-rate limits) that might delay signal and response^114^. We analyzed datasets for studies of two enzyme systems, and once again found that length of lag phase in some cases is inconsistent with, and in other cases correlates with steady-state rate (Fig. 9a,b) and oxidation rate (Fig. 9c) (Fig. 9)^115,116^. For [9-HPOD] and [13-HPOD] (metabolites produced under stress) (from *Terfezia claveryi* Chatin), there was no apparent correlation (r^2^ = 0.105, 0.043; Fig. 9a,b). By contrast, for mushroom tyrosine (EC 1.14.18.1) there was a strong correlation between length of lag phase and oxidation rate (Fig. 9c) (r^2^ = 0.956; Fig. 9c). However, more work is needed to determine whether findings such as those reported here occur across all the levels of complexity, even though the underlying mechanisms in each case may differ.
2. Studies of halophile communities (and other microbes found in extreme environments) suggest that lag phase in natural ecosystems can be prolonged. In such communities, it is possible that cells may, under some circumstances, experience lag phase for an indeterminate period. There is currently a paucity of data to demonstrate prolonged lag phases which progress at an infinitesimally slow rate. However, we hypothesize that extended lag phases are commonplace for the majority of the microbes in Earth’s biosphere and that, like for *in-vitro* cultures, lag phase is a measure of adaptation rather than stress *per se* for their *in-situ* populations.
3. The findings also raise questions about the meaning of ‘stress’ in the context of cellular systems, as well as the way we identify or measure it^13^. Batches of cells of a given microbe with different phenotypes (in different physiological conditions) likely respond differently from each other during lag phase. Also, the combination of conditions in which they existed and the new conditions that have triggered lag, will determine which changes occur in the cell. It may be, therefore, that molecules sometimes considered biomarkers, are reliable indicators of lag because they depend on cellular history, past environmental conditions, and new environmental conditions. Further work, including nuanced experimentation, is required to disentangle these issues in relation to potential biomarkers. The microbial lag phase can be easily identified and determined from a basic growth curve, and yet complexities arising from this simple parameter continue to generate scientific miscalculations in studies of microbiology.

## Experimental procedures

### *Bacillus subtilis* and *Escherichia coli* strains, nutrient medium, and culture conditions

A culture of *Bacillus subtilis* 168 (also known as DSM 402 or ATCC 23857) was obtained from M. Hecker of the University of Greifswald (Germany), and maintained on Belitzki minimal medium (15 mM [NH_4_]_2_SO_4_, 8 mM MgSO_4_:7H_2_0, 27 mM KCl, 7 mM Na_3_-citrate:2H_2_O, 50 mM tris-HCl, 0.6 mM KH_2_PO_4_, 2 mM CaCl_2_:2H_2_O, 1 µM FeSO_4_:7H_2_O, 10 µM MnSO_4_:4H_2_0, 11.1 mM glucose, 62.4 µM tryptophan and 4.5 mM glutamic acid) at 37 °C. The pH of the medium was buffered pre-sterilisation to 7.5 using KOH, HCl and NaOH. The pH was measured post-sterilisation using a Mettler Toldeo Seven Easy, pH probe (Mettler Toldeo; Zurich, Switzerland).

A culture of *Escherichia coli* BL21 (syn. DE3) was obtained from New England Biolabs. (Hitchin, UK) and maintained in Luria–Bertani (LB) broth (per litre: 10 g yeast extract and 5 g peptone [peptic digest from meat; Fluka Analytical]; pH 6.1) at 25 °C. The pH was measured post-sterilisation using Pehanon pH test strips (Macherey-Nagel; Düren, Germany).

### Culture of *B. subtilis* and *E. coli* under solute-induced stresses

Growth rates for *B. subtilis* 168 were determined in Belitzki minimal medium (control) and modified Belitzki minimal media, which had been supplemented with a range of concentrations of the following compounds: betaine, guanidine hydrochloride, ammonium sulphate, polyethylene glycol 600, glycerol, proline, glucose, MgCl_2_, sucrose, and polyethylene glycol 6000 (Table S4). All media were sterilised, using 500-ml aliquots in 1-litre glass Schott Duran bottles which were immersed in a water bath for 60 minutes at 100 °C, following which they were allowed to cool to 37 °C. For each medium, water activity was then determined, as described below, and values are given in Table S4. Aliquots (50 ml) of each medium were put into 250-ml conical flasks, which were then sealed with non-absorbent cotton wool. An exponential phase *B. subtilis* culture, grown in Belitzki minimal medium, was used to inoculate each medium to give a starting optical density_500 nm_ of ∼0.05. Cultures were incubated at 37 °C with shaking at 180 revolutions per minute (rpm). Optical densities_500 nm_ were determined every 40 minutes (or as required), and all treatments were carried out in triplicate.

Growth rates for *E. coli* BL21 (syn. DE3) were determined using cultures grown in LB broth (control) and LB broth supplemented with ethanol, butanol and urea over a range of concentrations (Table S4). Media were autoclaved, cooled to 23 °C and then ethanol or butanol was added using a Gilson Pipetman micropipette with the pipette tip inserted beneath the surface of the broth. Urea was weighed into a sterile vessel before being added to the media and swirled to dissolve. For each medium, pH was determined using Pehanon pH test strips and found to be between 5.9 and 6.1. Aliquots of each medium (50 ml) were placed into 100-ml Supelco serum bottles (Sigma-Aldrich, Dorset, UK) and sealed with aluminium foil. An *E. coli* culture grown to exponential phase in LB broth was used to inoculate each medium at a starting optical density (OD)_560 nm_ of 0.01–0.02. Cultures were placed on a shaking incubator (120 rpm) at 20 °C and OD_560 nm_ was determined every 1–4 hours.

### Determinations of lag phase, and exponential growth rate, for *B. subtilis* and *E. coli*

Mean optical density values were plotted against incubation time for each stressor type (Figs. S7 and S11). Lag phase was determined as described above^7^, and exponential growth rates were calculated according to Pirt^83^. The relationship between lag phase and exponential growth rate was characterized, for each medium type, as described below.

### Extremophilic fungal strains, nutrient media, and culture conditions

*Eurotium repens* JH06JPD were isolated by Williams and Hallsworth^51^ and are available from the corresponding author of the current article. The fungal xerophiles *Eurotium amstelodami* FRR 2792, *Eurotium echinulatum* FRR 5040, *Eurotium halophilicum* FRR 2471, *Xerochrysium xerophilum* FRR 0530, and *Xeromyces bisporus* FRR 0025, FRR 1522, FRR 2347, and FRR 3443 were obtained from CSIRO Food and Nutritional Sciences Culture Collection (North Ryde, NSW, Australia). Please note that *E. echinulatum*, *E. halophilicum*, and *E. repens* have recently been renamed as *Aspergillus brunneus*, *Aspergillus halophilicus* and *Aspergillus pseudoglaucus*, respectively^117^. Cultures were maintained on malt-extract yeast-extract phosphate agar (MYPiA; 1% w/v malt-extract (Oxoid; Hampshire, UK), 1% w/v yeast extract (Oxoid; Hampshire, UK), 1.5% w/v agar (Acros; Loughborough, UK), and 0.1% w/v anhydrous K_2_HPO_4_)supplemented with glycerol (5.5 M; 0.821 water activity) at 30 °C, as for previous studies^16,17,49^.

### Assays of fungal germination at low water-activity

For each xerophile strain, ability to germinate was assessed at biologically hostile water activities (0.765–0.570) previously, using a range of 36 culture media designed to recreate physicochemical stresses experienced by microbes in natural and anthropogenic systems^16^. They were based on MYPiA, but supplemented with glycerol + NaCl; glycerol + sucrose; glycerol + glucose + fructose; glycerol only; glycerol + NaCl + sucrose; and glycerol + NaCl + KCl + sucrose (Table S3). Generally, media were sterilized by autoclaving at 121 °C at 1 atm. For those supplemented with glucose + fructose, however, 500 ml aliquots of medium were instead placed into a 1-litre glass Schott Duran bottle (Sigma-Aldrich, Dorset, UK), and immersed in a water bath at 80 °C for 30 minutes. Regardless of the sterilization method, media were allowed to cool before pouring into Petri plates. Once media had solidified, water activity was determined as described below.

MYPiA + glycerol (5.5 M) medium was inoculated using 2-mm-diameter plugs of agar taken from the periphery of exponential phase cultures growing on medium of the same composition, and plates were placed in a sealed bag of low-density polyethylene to maintain a constant relative humidity (thus maintaining water activity), while allowing gaseous exchange. For strains of *A. penicillioides*, *E. amstelodami, E. echinulatum* and *E. repens*, plates were incubated for 10–14 days at 30 °C; for strains of *X. bisporus, X. xerophilum* and *E. halophilicum*, plates were incubated for 21–28 days at 30 °C. Spores were then harvested by covering Petri plates with sterile solutions of 5.5 M glycerol (15 ml) and aerial spores were dislodged by gently brushing with a sterile glass rod. The spore suspensions were then passed through sterile glass-wool twice to remove hyphal fragments as described in earlier studies^73,76^. Each suspension was adjusted, by adding sterile 5.5 M glycerol solution, to a final spore concentration of 1 × 10^6^ spores ml^−1^.

Spore suspension (150 µl) was used to inoculate each germination-assay medium by pipetting onto the agar surface and then spreading using a sterilize glass spreader. Plates of each type of medium were incubated in sealed bag of low-density polyethylene to maintain a constant relative humidity (in the dark; 30 °C). Periodically, a 4-mm agar disc was removed and used to immediately quantify percentage germination, via counts of 200 spores, using a light microscope. Upon removal of the disc, plates were resealed and placed back in the incubator. Germination was recorded for spores with a germ tube that was longer than the spore diameter, for isolated spores only (those located in clumps were not included in the assessment), as described previously^76,118^. These examinations were carried out at least daily, throughout a 50-d period, and all measurements were carried out in triplicate. These data were analysed as described below.

### Determinations of lag phase before, and rates of, fungal germination

Percentage germination was plotted against incubation time by Stevenson *et al*.^16^ (Fig. S1). The length of the lag phase was determined by extrapolating trend lines to a point of 0% spore germination for each media type, as described previously^16^. The plots of percentage germination versus time (Fig. S1) were also used to determine maximum rate of progress of germination; i.e. during the exponential phase (Fig. 4). The relationship between length of lag phase and germination rate was characterized, for each xerophile species, as described below.

### *Saccharomyces cerevisiae* strains, nutrient medium, and culture conditions

*Saccharomyces cerevisiae* strains (14 and 77) were obtained from the Yeast Strains Collection of Laboratory of Cell Biology, Institute of Microbiology and Biotechnology, University of Latvia. Each strain was maintained on yeast extract, peptone, dextrose agar (YPDA: 1% yeast extract, 2% peptone and 2% glucose and 2% w/v agar l^−1^) at 4 °C (Table S7). Each was cultivated in both a nutrient-rich, complex medium (yeast extract peptone dextrose broth; YPDB: 1% yeast extract, 2% peptone and 2% w/v glucose) and a salt-supplemented nutrient broth (SSNB: 0.7 g MgSO_4_, 0.5 g NaCl, 3.3 g (NH_4_)_2_SO_4_, 1 g KH_2_PO_4_, 0.13 g K_2_HPO_4_, 20 g glucose, and 3 g yeast extract l^−1^, adjusted to pH 5.5 by addition of KOH prior to autoclaving) at 23 or 30 °C (see below).

### *Saccharomyces cerevisiae* dehydration-rehydration experiments

For each strain, cells were inoculated into YPDB (50 ml in a 250-ml conical flask) and incubated in an orbital shaking incubator (180 rpm, 30 °C) for 24 h, by which time they were in the stationary growth phase (Table S7). A 0.5-ml aliquot was then removed from each flask and used to inoculate fresh YPDB or SSNB (100 ml in a 500-ml conical flask) and incubated (180 rpm, 30 °C) for 48 h. Cells were harvested by centrifugation at 2000 rpm (Eppendorf 5810 R, Hamburg, Germany) for 10 min. The supernatant was then discarded and the cell pellet was blotted with filter paper to remove any residual liquid. Following this, the pellet was pressed through a metal sieve (pore size = 1 mm), and then divided into two fractions. One was dried to a constant weight at 105 °C (±0.1 mg), using a fan-free oven. The other was placed on a piece of filter paper in a vented Petri plate and allowed to dehydrate by placing at 30 °C in the fan-free oven, until the water content of cells was reduced to 8–10% w/w according to the difference between the weights obtained at 105 °C and the initial weight. At this water content, the *S. cerevisiae* cells are known to enter a state of anhydrobiosis during which time a high level of viability is retained^119–121^.

The water content of these cells was reduced to 8–10% w/w (as described above). For determinations of cell viability after rapid rehydration, sterile distilled water or a 1 M xylitol solution (10 ml) was added to 50 mg of dried cells and shaken gently for 10 min at 20 °C^122^. Survival rates of dehydrated cultures were then determined by fluorescence microscopy. Five microlitres of 1 mg ml^−1^ stock of fluorochrome primuline (Sigma Aldrich) was added to 5 µl yeast suspension and placed on a microscope slide, under a cover slip, for assessment^123,124^. The percentage viable cells were determined by subtracting the number which appeared by eye to be bright under fluorescence (these were dead) from the total number that was observed under phase contrast. For each treatment there were two replicate samples and for each sample, a minimum of 600 cells were assessed. In some experiments gradual rehydration of dry cells in the vapour of water was performed. This was carried out by placing cells (50 mg) on parchment paper in a 3-l glass chamber (its volume 3 l) containing 200 ml of distilled water in the base (giving an equilibrium relative humidity of 100%) at 37 °C for 2 hours^122^. After this procedure, cells were subjected to a rapid rehydration, by suspending them into either distilled water or a 1 M xylitol solution for 10 mins.

### Determinations of lag phase, and rates of exponential growth, for *Saccharomyces cerevisiae* post-rehydration

After the rapid rehydration of cells, each suspension was standardized to OD_600_ = 0.5 by adding water and 10 µl was taken from each. These aliquots were used to inoculate YPDB or SSNB (190 µl) in a 96-well microplate at 30 °C for 24 h. Optical density_600 nm_ was then monitored in an Infinite M200Pro Microplate Reader (Tecan, Switzerland). Mean values of optical density were plotted against incubation time for each treatment as well as the control. Lag phase was determined according to Lodge and Hinshelwood^7^ (data not shown), and exponential growth rates were calculated according to Pirt^83^. The relationship between lag phase and exponential growth rate was characterized as described below.

### *Mrakia frigida* strains, nutrient medium, and culture conditions

*Mrakia frigida* strain DSM 70883 was obtained from DSMZ (Brunswick; Germany) and was cultured on MYPiA (0.997 water activity) at 1.7 °C^73^. Spot Test Assays were carried out at 1.7 °C: assessments of colony density (from 0 to 5 arbitrary units) were made at frequent intervals over a period of 210 days as described previously^52,73,125–129^. All Petri plates containing the same medium were sealed in a bag of low-density polyethylene to maintain a constant relative humidity (thus maintaining water activity), while allowing gaseous exchange. Experiments were carried out in triplicate, and plotted values are the means of independent treatments.

### Comparison with extant data for food-spoilage fungi and bacteria and enzyme systems

In order to determine whether the findings of the current study were applicable to mesophilic spoilage fungi and/or for enzyme systems, extant datasets were located, analyzed, and then compared with the model systems used in the current study. For the mesophilic fungi *Aspergillus aculeatinus* and *Aspergillus sclerotiicarbonarius*, that are known to cause food-spoilage, growth data^80^ were used to construct length of lag phase-versus-maximum growth-rate plots, as for *B. subtilis* (see above). As described by Akbar and Magan^80^, media was prepared by boiling 300 g of ground green coffee beans in 1 litre of distilled water for 30 min, then filtering through a double layer of muslin. All media contained 6% [w/v] concentrated coffee extract, agar (2% w/v) and glycerol; the latter was used to adjust water activity. Glycerol concentrations were 23, 9.7 or 2.7% w/v, and the water activites of these media were 0.900, 0.950 and 0.980, respectively. For the food-spoilage microbes, the mesophilic bacteria *E. faecalis*, *S. aureus* and *S. typhimurium* and the psychrotolerant bacteria *B. thermosphacta* and *P. fluorescens*, length of lag- and growth-rate data^87^ were used to construct length of lag phase-versus-maximum growth-rate plots, as described above for *B. subtilis*.

For *in-vitro* activity of lipoxygenase (extracted from the ascomycete *Terfezia claveryi*), the assay medium contained linoleic acid solution (10 µl from a stock solution of 18 mM, in ethanol), sodium borate buffer (0.1 M, pH 10), lipoxygenase (20 µl in 0.1 M sodium borate buffer at pH 10.0), and the hydroperoxy octadecadienoic acids (HPODs) 9-HPOD and 13-HPOD over a range of concentrations (0 to 19.3 µM); made up to a final volume of 1 ml. The length of the lag phase was quantified by Pérez-Gilabert *et al*.^116^ by measuring activity spectrophotometrically (234 nm) for a period of time to construct a reaction progress curve, constructing a straight line through the maximal rate section of this curve, and then determining the value at which this straight line intercepts the time axis. The steady-state rate was calculated according to the maximal rate of the reaction progress curve^116^. These data were used in the current study to construct plots values of length of lag phase versus steady-state rate of catalytic activity (Fig. 9a,b).

For *in-vitro* activity of tyrosinase (extracted from the basodiomycete *Agaricus bisporus*), the assay medium (3 ml) contained sodium phosphate buffer (50 mM, pH 6.8), anisic acid over a range of concentrations (0 to 4 mM), tyrosinase (0.1 ml from a 1.0 mg ml^−1^ stock solution in 0.1 M phosphate buffer, pH 6.8), and tyrosine (2 mM)^115^. Kubo *et al*.^115^ state that “0.1 ml of the sample solution and 0.1 ml of the aqueous solution of the mushroom tyrosinase (0.2 mg ml^−1^ in 0.1 M phosphate buffer, pH 6.8) were added in this order to the mixture. This solution was immediately monitored [at 30 °C]. The enzyme activity was monitored by dopachrome formation at 475 nm”, so absorbance (475 nm) was monitored continuously using a spectrophotometer^115^. The length of lag phase was also determined by Kubo *et al*.^115^. Oxidation rates were calculated in the current study using the absorbance measurements to detect dopachrome that is produced via the enzyme-catalyzed oxidation of the substrate^115^. Plots of length of lag phase versus oxidation rates were constructed (Fig. 9c).

### Characterization of relationship between microbial lag phases and stress

For microbial systems, maximum rates of growth or germination have traditionally been used as a reliable indicator for, and measure of, cellular stress (see above). For growth of *Bacillus*, *S. cerevisiae, M. frigida, E. coli* or mesophilic fungi, germination of fungal xerophiles, mesophilic bacteria, psychrotolerant bacteria and catalytic activity of fungal enzymes, length of lag phase was plotted against maximum rates (Figs. 1, 3–7, 9, S2, S8–S10 and S12). Using Microsoft Excel, 2016 MSO, Microsoft Corporation, California, USA, trend lines which provided the closest fit and highest regression coefficient (r^2^) value were added. Plots were fitted with linear, exponential, logarithmic, power- and polynomial (orders 2, 3, 4 and 5) trend lines and, in each case, the curve which best-described the data and gave the highest r^2^ value selected as described in earlier studies^16^. Values for r^2^ of >0.810 were considered to represent a strong correlation, those in the range 0.580 to 0.773 a moderately good correlation, those in the range 0.512 to 0.544 suggested a weak correlation, and those of <0.500 indicated inconsistency between lag phase and rate of growth/germination.

### Quantitation of water activity

For all culture media, water-activity values were determined empirically using a Novasina Humidat  IC-II water-activity machine fitted with an alcohol-resistant humidity sensor and eVALC alcohol filter (Novasina, Pfäffikon, Switzerland). The instrument was calibrated between each measurement using saturated salt solutions of known water activity^130^, and measurements were taken at the same temperature at which cultures were to be incubated. The water activity of each medium type was determined three times, and variation was within ±0.001. A number of additional precautions were taken to ensure accuracy of readings, as described previously^18,131^.

## Supplementary information

Supplementary Tables

Supplementary Figures
