# Supplementary material for: Microbial lag phase can be indicative of, or independent from, cellular stress
Source: Sci Rep. 2020 Apr 3;10:5948. doi: 10.1038/s41598-020-62552-4 (PMC7125082; doi:10.1038/s41598-020-62552-4)
Supplement: Supplementary file 1 — Supplementary Tables [file 41598_2020_62552_MOESM1_ESM.pdf]

Supplementary Information Tables

Microbial lag phase can be indicative of, or independent from, cellular stress

Philip G. Hamill[1], Andrew Stevenson[1], Phillip E. McMullan[1], James P. Williams[1], Abiann D. R. Lewis[1], Sudharsan S[2], Kath E. Stevenson[3], Keith D. Farnsworth[1], Galina Khroustalyova[4], Jon Y. Takemoto[5], John P. Quinn[1], Alexander Rapoport[4], and John E. Hallsworth[1]

[1]*Institute for Global Food Security, School of Biological Sciences, Queen's University Belfast, 19 Chlorine Gardens, Belfast, BT9 5DL, Northern Ireland.*
[2]*Department of Chemistry, PGP College of Arts and Science, NH-7, Karur Main Road, Paramathi, Namakkal, Tamil Nadu, 637 207, India*
[3]*Special Collections and Archives, McClay Library, Queen's University Belfast, 10 College Park Avenue, Belfast, BT7 1LP, Northern Ireland.*
[4]*Laboratory of Cell Biology, Institute of Microbiology and Biotechnology, University of Latvia, Jelgavas Str., 1-537, LV-1004 Riga, Latvia.*
[5]*Utah State University, Department of Biology, 5305 Old Main Hill Logan, UT 84322, USA.*

Supplementary Tables

Table S1. Model systems used in the current study.

Table S2. Culture conditions pre- and post-inoculation for microbes used in determinations of lag and germination- or growth rates.

Table S3. Media used for extremophilic fungal strain germination assays.

Table S4: Culture media used for the determination of lag phase of *Bacillus subtilis* and *Escherichia coli.*

Table S5. Biotic windows of model microorganisms in relation to solute stressors used in the current study.

Table S6. Relationship between lag phase and exponential rate for either planktonic growth of bacteria; germination of xerophilic fungi; de-/rehydration cycles of *S. cerevisiae*; germination of mesophilic fungi and enzyme systems.

Table S7. Dehydration-rehydration treatments and culture conditions for assays of *S. cerevisiae* strains.

Table S1. Model systems used in the current study.

Organism	Morphology/ structure	Propagules	Stress biology	Genomic status[a]	Number of published studies[b]
**Fungi**					
**Ascomycota**					
Aspergillus aculeatinus	Hyphal growth, producing uniseriate colonies	Conidia	Mesophilic	Whole genome sequenced	4[c]
Aspergillus penicillioides	Hyphal growth, producing uniseriate colonies	Conidia	Extremely xerophilic & halophilic*[18,132]	Genes & proteins have been sequenced	64[d]
Aspergillus sclerotiicarbonarius	Hyphal growth, producing biseriate colonies	Conidia	Mesophilic	Whole genome sequenced	2[e]
Eurotium amstelodami	Hyphal growth producing uniseriate colonies	Ascospores; conidia	Extremely xerophilic; halophilic[16,133]	Genes & proteins have been sequenced	174[f]
Eurotium echinulatum	Hyphal growth producing colonies	Ascospores; conidia	Extremely xerophilic[16]	Genes & proteins have been sequenced	4[g]
Eurotium halophilicum	Hyphal growth, producing colonies	Conidia	Extremely xerophilic; halotolerant[16]	Genes & proteins have been sequenced	13[h]
Eurotium repens	Hyphal growth, producing colonies	Ascospores; conidia	Extremely xerophilic; halophilic[16,133]	Genes have been sequenced	54[i]
Saccharomyces cerevisiae	Dispersed cells; pseudo-hyphal colonies	Ascospores	Poly-extremotolerant; resistant to desiccation-rehydration cycles[32]	Whole genome sequenced	147340[j]
Xerochrysium xerophilum	Hyphal growth, producing colonies	Arthroconidia; chlamydospores; conidia	Extremely xerophilic[16]	Genes & proteins have been sequenced	9[k]

Xeromyces bisporus	Hyphal growth, producing colonies	Aleurioconidia; ascospores	Extremely xerophilic[16]	Whole genome sequenced	17[l]
**Basidiomycota**					
Mrakia frigida	Hyphal growth, producing colonies	Basidiospores; teliospores	Psychrophilic	Whole genome sequenced	15[m]
**<u>Bacteria</u>**					
**Gram-negative**					
Escherichia coli	Rod-shaped cells	(non-spore-forming)	Mesophilic[134]	Whole genome sequenced	450956[n]
Pseudomonas fluorescens	Rod-shaped; elongated cells	(non-spore-forming)	Mesophilic[135]	Whole genome sequenced	10671[o]
Salmonella typhimurium	Rod-shaped cells	(non-spore-forming)	Mesophilic[134]	Whole genome sequenced	33525[p]
**Gram-positive**					
Bacillus subtilis	Matrix-producing cells; sporulating cells	Endospores	Thermophilic[136]	Whole genome sequenced	54772[q]
Brochothrix thermosphacta	Coccobacilli; rods; chain formation	(non-spore-forming)	Mesophilic	Whole genome sequenced	553[r]
Enterococcus faecalis	Cocci; short chain formation	(non-spore-forming)	Mesophilic	Whole genome sequenced	12588[s]
Staphylococcus aureus	Cocci; clustered	(non-spore-forming)	Mesophilic[134]	Whole genome sequenced	129533[t]
**<u>Macromolecular (enzyme-) systems</u>**					
Lipoxygenase	Non-haem iron-containing	(not applicable)	(not applicable)	Amino acid sequence	4[u]

(extracted from *Terfezia claveryi*)	enzyme[116]			determined for lipoxygenase from [*Solanum lycopersicum*]	
Tyrosinase (extracted from *Agaricus bisporus)*	Binuclear two-copper enzyme[137]	(not applicable)	(not applicable)	Amino acid sequence determined for *Agaricus bisporus* tyrosinase	310[v]

a. Data obtained from the NCBI Genome, Nucleotide and Protein database (05 February 2020).
b. Data obtained from the Thomson Reuters Web-of-Science database (05 February 2020).
c. According to a Web-of-Science topic search: “*Aspergillus-aculeatinus*”
d. According to a Web-of-Science topic search: “*Aspergillus-penicillioides* or *Aspergillus-penicilloides”*
e. According to a Web-of-Science topic search: “*Aspergillus-sclerotiicarbonarius”*
f. According to a Web-of-Science topic search: “*Eurotium-amstelodami* or *Aspergillus-amstelodami*”
g. According to a Web-of-Science topic search: “*Eurotium-echinulatum* or *Aspergillus-echinulatus* or *Aspergillus-brunneus*”
h. According to a Web-of-Science topic search: “*Eurotium-halophilicum* or *Aspergillus halophilicus*”
i. According to a Web-of-Science topic search: “*Eurotium-repens*”
j. According to a Web-of-Science topic search: “*Saccharomyces-cerevisiae*”
k. According to a Web-of-Science topic search: “*Xerochrysium-xerophilum*” “*Chrysosporium- xerophilum*”
l. According to a Web-of-Science topic search: “*Xeromyces-bisporus* or *Monascus-bisporus*”
m. According to a Web-of-Science topic search: “*Mrakia-frigida*”
n. According to a Web-of-Science topic search: “*Escherichia-coli*”
o. According to a Web-of-Science topic search: “*Pseudomonas-fluorescens*”
p. According to a Web-of-Science topic search: “*Salmonella-typhimurium*”
q. According to a Web-of-Science topic search: “*Bacillus-subtilis*”
r. According to a Web-of-Science topic search: “*Brochothrix-thermosphacta*”
s. According to a Web-of-Science topic search: “*Enterococcus-faecalis*”
t. According to a Web-of-Science topic search: “*Staphylococcus-aureus*”
u. According to a Web-of-Science topic search: “*Terfezia-claveryi* and (LOX or lipoxygenase)”
v. According to a Web-of-Science topic search: “*Agaricus-bisporus* and tyrosinase”

*capable of differentiation and cell division at lower water activity than any other microbe[17].

Table S2. Media used for extremophilic fungal strain germination assays[a,b].

Stressor type and concn. (M)	pH	Chao-/kosmo-tropicity (kJ g^{-1})	Water activity[c]
glycerol (5.50) + NaCl (0.50)	6.7	1.41	0.765
glycerol (5.50) + NaCl (1.0	6.8	0.66	0.741
glycerol (5.50) + sucrose (0.25)	6.6	12.45	0.734
glycerol (5.50) + NaCl (1.50)	6.7	-4.15	0.709
glycerol (7.00)	6.3	18.43	0.707
glycerol (5.50) + NaCl (0.50) + sucrose (0.30)	6.7	1.11	0.701
glycerol (5.50) + sucrose (0.50)	6.5	10.95	0.699
glycerol (5.50) + glucose (0.80) + fructose (0.80)	5.6	18.92	0.694
glycerol (5.50) + NaCl (1.60)	6.8	-5.15	0.692
glycerol (5.50) + NaCl (0.50) + sucrose (0.50)	6.7	-0.62	0.685
glycerol (5.50) + sucrose (0.65)	6.5	9.13	0.674
glycerol (5.50) + NaCl (1.70)	6.8	-6.49	0.668
glycerol (7.10)	6.3	19.59	0.664
glycerol (7.20)	6.2	21.58	0.654
glycerol (5.50) + NaCl (0.80) + sucrose (0.50)	6.6	-1.12	0.651
glycerol (5.50) + glucose (1.00) + fructose (1.00)	5.4	20.75	0.649
glycerol (7.30)	6.2	22.36	0.647
glycerol (5.50) + NaCl (1.80)	6.7	-11.04	0.640
glycerol (5.50) + NaCl (0.80) + sucrose (0.50) + KCl (0.20)	7.0	-2.14	0.639
glycerol (5.50) + sucrose (0.80)	6.3	7.30	0.637
glycerol (7.40)	6.2	22.83	0.635
glycerol (5.50) + NaCl (1.90)	6.7	-12.45	0.628
glycerol (5.50) + NaCl (1.00) + sucrose (0.70)	6.5	-4.31	0.623
glycerol (7.50)	6.2	26.56	0.621
glycerol (5.50) + sucrose (0.95)	6.3	5.39	0.619
glycerol (5.50) + glucose (1.25) + fructose (1.25)	5.4	22.74	0.611
glycerol (7.60)	6.1	27.64	0.608
glycerol (5.50) + NaCl (2.00)	6.5	-14.94	0.605
glycerol (5.50) + NaCl (0.80) + sucrose (0.80) + KCl (0.20)	6.8	-5.38	0.602
glycerol (5.50) + sucrose (1.10)	6.3	2.69	0.601
glycerol (5.50) + NaCl (1.20) + sucrose (0.80)	6.3	-6.52	0.598
glycerol (5.50) + glucose (1.50) + fructose (1.50)	5.3	25.49	0.592
glycerol (5.50) + sucrose (1.30)	6.2	0.65	0.591
glycerol (7.70)	6.1	29.05	0.585
glycerol (5.50) + NaCl (1.00) + sucrose (0.80) + KCl (0.20)	7.0	-6.68	0.579
glycerol (5.50) + NaCl (2.30)	6.6	-24.90	0.575

[a]Modified from Stevenson *et al.*[16].
[b]All media were based on MYPiA[51], and contained: 1% malt extract, 1% yeast extract, 0.1% KH_2PO_4, and 1.5% (w/v) agar.
[c]The water activity of each medium was measured at 30ºC, and replicate values were within ±0.002 water activity (see *Experimental procedures)*.

Table S3. Media used for extremophilic fungal strain germination assays[a,b].

Stressor type and concn. (M)	pH	Chao-/kosmo-tropicity (kJ g^{-1})	Water activity[c]
glycerol (5.50) + NaCl (0.50)	6.7	1.41	0.765
glycerol (5.50) + NaCl (1.0	6.8	0.66	0.741
glycerol (5.50) + sucrose (0.25)	6.6	12.45	0.734
glycerol (5.50) + NaCl (1.50)	6.7	-4.15	0.709
glycerol (7.00)	6.3	18.43	0.707
glycerol (5.50) + NaCl (0.50) + sucrose (0.30)	6.7	1.11	0.701
glycerol (5.50) + sucrose (0.50)	6.5	10.95	0.699
glycerol (5.50) + glucose (0.80) + fructose (0.80)	5.6	18.92	0.694
glycerol (5.50) + NaCl (1.60)	6.8	-5.15	0.692
glycerol (5.50) + NaCl (0.50) + sucrose (0.50)	6.7	-0.62	0.685
glycerol (5.50) + sucrose (0.65)	6.5	9.13	0.674
glycerol (5.50) + NaCl (1.70)	6.8	-6.49	0.668
glycerol (7.10)	6.3	19.59	0.664
glycerol (7.20)	6.2	21.58	0.654
glycerol (5.50) + NaCl (0.80) + sucrose (0.50)	6.6	-1.12	0.651
glycerol (5.50) + glucose (1.00) + fructose (1.00)	5.4	20.75	0.649
glycerol (7.30)	6.2	22.36	0.647
glycerol (5.50) + NaCl (1.80)	6.7	-11.04	0.640
glycerol (5.50) + NaCl (0.80) + sucrose (0.50) + KCl (0.20)	7.0	-2.14	0.639
glycerol (5.50) + sucrose (0.80)	6.3	7.30	0.637
glycerol (7.40)	6.2	22.83	0.635
glycerol (5.50) + NaCl (1.90)	6.7	-12.45	0.628
glycerol (5.50) + NaCl (1.00) + sucrose (0.70)	6.5	-4.31	0.623
glycerol (7.50)	6.2	26.56	0.621
glycerol (5.50) + sucrose (0.95)	6.3	5.39	0.619
glycerol (5.50) + glucose (1.25) + fructose (1.25)	5.4	22.74	0.611
glycerol (7.60)	6.1	27.64	0.608
glycerol (5.50) + NaCl (2.00)	6.5	-14.94	0.605
glycerol (5.50) + NaCl (0.80) + sucrose (0.80) + KCl (0.20)	6.8	-5.38	0.602
glycerol (5.50) + sucrose (1.10)	6.3	2.69	0.601
glycerol (5.50) + NaCl (1.20) + sucrose (0.80)	6.3	-6.52	0.598
glycerol (5.50) + glucose (1.50) + fructose (1.50)	5.3	25.49	0.592
glycerol (5.50) + sucrose (1.30)	6.2	0.65	0.591
glycerol (7.70)	6.1	29.05	0.585
glycerol (5.50) + NaCl (1.00) + sucrose (0.80) + KCl (0.20)	7.0	-6.68	0.579
glycerol (5.50) + NaCl (2.30)	6.6	-24.90	0.575

[a]Modified from Stevenson *et al.*[16].
[b]All media were based on MYPiA[51], and contained: 1% malt extract, 1% yeast extract, 0.1% KH_2PO_4, and 1.5% (w/v) agar.
[c]The water activity of each medium was measured at 30ºC, and replicate values were within ±0.002 water activity (see *Experimental procedures)*.

Table S4. Culture media used for the determination of lag phase of *Bacillus subtilis* and *Escherichia coli.*

Solute	Chaotrope-induced osmotic stress	Water activity range	Stressor concentration for each series of media[a]						
			A	B	C	D	E	F	G
Bacillus subtilis:									
Glycerol	Neutral	0.998-0.941	2.71	2.26	1.81	1.36	0.90	0.45	0.00 M
Glucose	Osmotic	0.996-0.956	1.94M	1.62	1.30	0.97	0.65	0.32	0.00 M
$MgCl_2$	Chaotropic	0.995-0.969	1.37M	1.14	0.91	0.68	0.46	0.23	0.00 M
Proline	Kosmotropic	0.993-0.932	2.61M	2.17	1.74	1.30	0.87	0.43	0.00 M
NH_2SO_4	Kosmotropic	0.995-0.945	1.51M	1.26	1.01	0.76	0.50	0.25	0.00 M
Sucrose	Osmotic	Not Measured	1.31	1.10	0.88	0.66	0.44	0.22	0.00 M
Betaine	Neutral	0.995-0.914	2.56	2.13	1.71	1.28	0.85	0.43	0.00 M
PEG-6000	Matric	0.998-0.988	50.00	41.67	33.33	25.00	16.67	8.33	0.00 mM
gnHCL	Chaotropic	0.998-0.995	150.00	125.00	100.00	75.00	50.00	25.00	0.00 mM
PEG-600	Osmotic	0.999-0.987	400.00	333.33	266.67	200.00	133.33	66.67	0.00 mM
Escherichia coli:									
Ethanol	Chaotropic	Not Measured	850.0	800.0	750.0	700.0	650.0	600.0	0.00 nM
Butanol	Chaotropic	Not Measured	120.00	110.0	100.0	90.0	80.0	70.00 nM	
Urea	Chaotropic	Not Measured	900.0	800.0	700.0	600.0	500.0	400.0	300.0 nM

[a]Values are stated using molar (M), millimolar (mM) or nanomolar (nM) units.

Table S5. Biotic windows of model microorganisms (Table S1) in relation to solute stressors used in the current study.

Solute(s)	Window for growth or germination
**Bacterium**	
Bacillus subtilis 168 (syn. DSM 402; ATCC 23857)	*Windows for growth*
Glycerol	Up to 2.70 M (Fig. S2a)
Proline	Up to 2.60 M (Fig. S2b)
Glucose	Up to 1.90 M (Fig. S2c)
$MgCl_2$	Up to 1.40 M (Fig. S2d)
Sucrose	Up to 1.30 M (Fig. S2e)
Ammonium sulfate	Up to 0.80 M (Fig. S7f)
Betaine	Up to 2.60 M (Fig. S7a)
Guanidine hydrochloride	Up to 150 mM (Fig. S7b)
Polyethylene glycol 6000	Up to 50 mM (Fig. S7c)
Polyethylene glycol 600	Up to 400 mM (Fig. S7d)
NaCl	Up to 1140 mM[54]
Urea	Up to 755 mM[54]
Escherichia coli BL21 (syn. DE3)	*Windows for growth* (Fig. S6)
Ethanol	Up to 850 mM (theoretical maximum 931 mM; Fig. S6)
Butanol	Up to 120 mM
Urea	Up to 837 mM (theoretical maximum >853 mM; Fig. S6)
**Fungi**	
Aspergillus penicillioides JH06GBM	*Windows for germination* (Fig. S13a)
Glycerol	Up to 7.20 M
Glycerol (5.5 M) + NaCl	Glycerol (5.5 M) + up to 1.80 M NaCl
Glycerol (5.5 M) + sucrose	Glycerol (5.5 M) + up to 0.65 M sucrose
Glycerol (5.5 M) + NaCl + sucrose (0.5 M)	Glycerol (5.5 M) + sucrose (0.5 M) up to 0.80 M NaCl
Glycerol (5.5 M) + glucose + fructose	Glycerol (5.5 M) + up to 0.80 M glucose + 0.80 M fructose
Aspergillus penicillioides JH06THH	*Windows for germination* (Fig. S1a)
Glycerol	Up to 7.10 M
Glycerol (5.5 M) + NaCl	Glycerol (5.5 M) + up to 1.60 M NaCl
Glycerol (5.5 M) + sucrose	Glycerol (5.5 M) + up to 0.65 M sucrose
Glycerol (5.5 M) + NaCl (0.5 M) + sucrose	Glycerol (5.5 M) + NaCl (0.5 M) + up to 0.50 M sucrose
A. penicillioides JH06THJ (syn. FRR 6206)	*Windows for germination* (Fig. S13b)
Glycerol (5.5 M) + NaCl	Glycerol (5.5 M) + up to 1.90 M NaCl
Glycerol (5.5 M) + sucrose	Glycerol (5.5 M) + up to 0.65 M sucrose
Glycerol (5.5 M) + NaCl + sucrose	Glycerol (5.5 M) + up to 0.80 M NaCl + 0.50 M sucrose
Eurotium amstelodami FRR 2792	*Windows for germination* (Fig. S13c)
Glycerol (5.5 M) + NaCl	Glycerol (5.5 M) + up to 1.60 M NaCl
Glycerol (5.5 M) + sucrose	Glycerol (5.5 M) + up to 0.50 M sucrose
Glycerol (5.5 M) + NaCl (0.5 M) + sucrose	Glycerol (5.5 M) + NaCl (0.5 M) + up to 0.50 M sucrose
Glycerol (5.5 M) + glucose + fructose	Glycerol (5.5 M) + up to 0.80 M glucose + 0.80 M fructose
Eurotium echinulatum FRR 5040	*Windows for germination* (Fig. S13d)
Glycerol (5.5 M) + NaCl	Glycerol (5.5 M) + up to 1.00 M NaCl

Glycerol (5.5 M) + sucrose	Glycerol (5.5 M) + up to 0.65 M sucrose
Glycerol (5.5 M) + NaCl (0.5 M) + sucrose	Glycerol (5.5 M) + NaCl (0.5 M) + up to 0.30 M sucrose
Glycerol (5.5 M) + glucose + fructose	Glycerol (5.5 M) + up to 0.80 M glucose + 0.80 M fructose
Eurotium halophilicum FRR 2471	*Windows for germination* (Fig. S13e)
Glycerol (5.5 M) + NaCl	Glycerol (5.5 M) + up to 1.80 M NaCl
Glycerol (5.5 M) + sucrose	Glycerol (5.5 M) + up to 0.65 M sucrose
Glycerol (5.5 M) + NaCl + sucrose	Glycerol (5.5 M) + up to 0.80 M NaCl + 0.50 M sucrose
Eurotium repens JH06JPD	*Windows for germination* (Fig. S13f)
Glycerol (5.5 M) + NaCl	Glycerol (5.5 M) + up to 1.50 M NaCl
Glycerol (5.5 M) + sucrose	Glycerol (5.5 M) + up to 0.50 M sucrose
Glycerol (5.5 M) + NaCl (0.5 M) + sucrose	Glycerol (5.5 M) + NaCl (0.5 M) + up to 0.50 M sucrose
Mrakia frigida DSM 70883	*Windows for growth* (Fig. S5)
Sucrose	Up to 1.50 M
Glucose	Up to 2.80 M
Glycerol	Up to 4.30 M
NaCl	Up to 2.50 M
$MgCl_2$	Up to 1.00 M
Xerochrysium xerophilum FRR 0530	*Windows for germination* (Fig. S1d)
Glycerol	Up to 7.10 M
Glycerol (5.5 M) + NaCl	Glycerol (5.5 M) + up to 1.70 M NaCl
Glycerol (5.5 M) + sucrose	Glycerol (5.5 M) + up to 0.50 M sucrose
Glycerol (5.5 M) + NaCl (0.5 M) + sucrose	Glycerol (5.5 M) + NaCl (0.5 M) + up to 0.50 M sucrose
Glycerol (5.5 M) + glucose + fructose	Glycerol (5.5 M) + up to 0.80 M glucose+ 0.80 M fructose
Xeromyces bisporus FRR 0025	*Windows for germination* (Fig. S13g)
Glycerol	Up to 7.10 M
Glycerol (5.5 M) + NaCl	Glycerol (5.5 M) + up to 0.50 M NaCl
Glycerol (5.5 M) + sucrose	Glycerol (5.5 M) + up to 0.80 M sucrose
Glycerol (5.5 M) + NaCl (0.5 M) + sucrose	Glycerol (5.5 M) + NaCl (0.5 M) + up to 0.50 M sucrose
Glycerol (5.5 M) + glucose + fructose	Glycerol (5.5 M) + up to 1.00 M glucose + 1.00 M fructose
X. bisporus FRR 1522	*Windows for germination* (Fig. S1c)
Glycerol (5.5 M) + NaCl	Glycerol (5.5 M) + up to 1.60 M NaCl
Glycerol (5.5 M) + sucrose	Glycerol (5.5 M) + up to 0.80 M sucrose
Glycerol (5.5 M) + NaCl (0.5 M) + sucrose	Glycerol (5.5 M) + NaCl (0.5 M) + up to 0.50 M sucrose
Glycerol (5.5 M) + glucose + fructose	Glycerol (5.5 M) + up to 1.00 M glucose + 1.00 M fructose
X. bisporus FRR 2347	*Windows for germination* (Fig. S13g)
Glycerol (5.5 M) + NaCl	Glycerol (5.5 M) + up to 1.60 M NaCl
Glycerol (5.5 M) + sucrose	Glycerol (5.5 M) + up to 0.80 M sucrose
Glycerol (5.5 M) + NaCl (0.5 M) + sucrose	Glycerol (5.5 M) + NaCl (0.5 M) + up to 0.50 M sucrose
Glycerol (5.5 M) + glucose + fructose	Glycerol (5.5 M) + up to 1.00 M glucose + 1.00 M fructose
X. bisporus FRR 3443	*Windows for germination* (Fig. S1b)

Glycerol (5.5 M) + NaCl	Glycerol (5.5 M) + up to 1.60 M NaCl
Glycerol (5.5 M) + sucrose	Glycerol (5.5 M) + up to 0.80 M sucrose
Glycerol (5.5 M) + NaCl (0.5 M) + sucrose	Glycerol (5.5 M) + NaCl (0.5 M) + up to 0.50 M sucrose
Glycerol (5.5 M) + glucose + fructose	Glycerol (5.5 M) + up to 0.80 M glucose + 0.80 M fructose

Table S6. Relationship between lag phase and exponential rate for either planktonic growth of bacteria (Figs. 1; S2; S10 and S12); germination of xerophilic fungi (Figs. 4 and S8); de-/rehydration cycles of *S. cerevisiae* (Figs. 5 and 6); germination of mesophilic fungi (Figs. 3 and 7) and enzyme systems (Fig. 8).

Microorganism and strain (figure)	Stressor used to supplement culture medium	Trend line which best fits the data[a]	Equation of trend line[b]	Gradient of trend line (units)[c]
Bacteria				
Bacillus subtillus				
168 (Fig. 1a)	Betaine	Linear	$y = -156.57x + 43.669$	-156.57
168 (Fig. 1b)	Guanidine hydrochloride	Linear	$y = -26.493x + 21.252$	-26.493
168 (Fig. 1c)	Polyethylene glycol 6000	Power	$y = 1.1903x^{-0.663}$	N/A
168 (Fig. 1d)	Polyethylene glycol 600	Polynomial order 3	$y = -0.0002x^3 + 0.0295x^2 – 1.9839x + 50.167$	N/A
168 (Fig. S2a)	Glycerol	Power	$y = -0.2174x^{-1.525}$	N/A
168 (Fig. S2b)	Proline	Power	$y = 1.344x^{-1.231}$	N/A
168 (Fig. S2c)	Glucose	Power	$y = 172.26x^{-0.849}$	N/A
168 (Fig. S2d)	$MgCl_2$	Polynomial order 3	$y = -4E-05x^3 + 0.0101x^2 – 0.769x + 19.76$	N/A
168 (Fig. S2e)	Sucrose	Polynomial order 4	$y = 3E-06x^4 - 0.0004x^3 + 0.0333x^2 - 1.3236x + 24.817$	N/A
168 (Fig. S2f)	Ammonium sulphate	Polynomial order 2	y = -16.087x2 + 1.4361x + 7.6602	N/A
Brochothrix thermosphacta				
ATCC 12706 (Fig. S12b)	NaCl	Power	$y = 1.6228x^{-1.02}$	N/A
ATCC 12706 (Fig. S12g)	Glycerol	Power	$y = 0.3977x^{-1.433}$	N/A

ATCC 12706 (Fig. S12l)	Sucrose	Exponential	$y = 46.181e^{-7.678x}$	N/A
Enterococcus faecalis				
ATCC 7080 (Fig. S12c)	NaCl	Power	$y = 0.7831x^{-0.917}$	N/A
ATCC 7080 (Fig. S12h)	Glycerol	Power	$y = 0.5582x^{-0.665}$	N/A
ATCC 7080 (Fig. S12m)	Sucrose	Power	$y = 1.8843x^{-1.016}$	N/A
Escherichia coli				
BL21 D3 (Fig. S10a)	Ethanol	Polynomial order 2	y = 0.0745x2 - 2.1971x + 21.395	N/A
BL21 D3 (Fig. S10b)	Butanol	Power	$y = 31.317x^{-0.497}$	N/A
BL21 D3 (Fig. S10c)	Urea	Polynomial order 2	y = 0.0193x2 - 0.8843x + 16.067	N/A
Pseudomonas fluorescens				
ATCC 17400 (Fig. S12a)	NaCl	Exponential	y = 66.962e-7.019x	N/A
ATCC 17400 (Fig. S12f)	Glycerol	Power	$y = 0.1875x^{-1.415}$	N/A
ATCC 17400 (Fig. S12k)	Sucrose	Power	$y = 2.3433x^{-1.061}$	N/A
Salmonella typhimurium				
ATCC 13311 (Fig. S12d)	NaCl	Power	$y = 1.1215x^{-1.098}$	N/A
ATCC 13311 (Fig. S12i)	Glycerol	Power	$y = 0.1657x^{-1.686}$	N/A
ATCC 13311 (Fig. S12n)	Sucrose	Power	$y = 4.3751x^{-0.635}$	N/A
Staphylococcus aureus				
ATCC 13566 (Fig. S12e)	NaCl	Power	$y = 0.9828x^{-1.325}$	N/A
ATCC 13566 (Fig. S12j)	Glycerol	Power	$y = 0.5805x^{-1.108}$	N/A
ATCC 13566 (Fig. S12o)	Sucrose	Power	$y = 1.7044x^{-0.939}$	N/A

Xerophilic fungi				
Aspergillus penicillioides				
JH06THH (Fig. 4a)	Glycerol or glycerol plus other stressor(s)	Linear	$y = -3.8183x + 17.382$	-3.8183
JH06GBM (Fig. S8a)	Glycerol or glycerol plus other stressor(s)	Linear	$y = -7.2162x + 16.856$	-7.2162
JH06THJ (Fig. S8b)	Glycerol or glycerol plus other stressor(s)	Exponential	$y = 28.516e^{-0.937x}$	N/A
Eurotium amstelodami				
FRR 2792 (Fig. S8c)	Glycerol or glycerol plus other stressor(s)	Polynomial order 2	$y = 4.1057x^2 - 17.989x + 24.425$	N/A
Eurotium echinulatum				
FRR 5040 (Fig. S8d)	Glycerol or glycerol plus other stressor(s)	Linear	$y = -4.7651x + 14.372$	-4.7651
Eurotium halophilicum				
FRR 2471 (Fig. S8e)	Glycerol or glycerol plus other stressor(s)	Linear	$y = -12.139x + 22.315$	-12.139
Eurotium repens				
JH06JPD (Fig. S8f)	Glycerol or glycerol plus other stressor(s)	Linear	$y = -10.922x + 24.242$	-10.922
Xeromyces bisporous				
FRR 3443 (Fig. 4b)	Glycerol or glycerol plus other stressor(s)	Linear	$y = -1.4468x + 11.434$	-1.4468

FRR 1522 (Fig. 4c)	Glycerol or glycerol plus other stressor(s)	Polynomial order 3	$y = 14.287x^3 - 40.514x^2 + 19.335x + 18.622$	N/A
FRR 0025 (Fig. S8g)	Glycerol or glycerol plus other stressor(s)	Linear	$y = -9.3797x + 21.154$	-9.3797
FRR 2347 (Fig. S8h)	Glycerol or glycerol plus other stressor(s)	Exponential	$y = 29.19e^{-0.944x}$	N/A
Xerochrysium xerophilium				
FRR 0530 (Fig. 4d)	Glycerol or glycerol plus other stressor(s)	Polynomial order 4	$y = 28.017x^4 - 153.21x^3 + 302.36x^2 - 258.22x+ 87.801$	N/A
<u>Yeast</u>				
Saccharomyces cerevisiae				
23°C				
14 (Fig. 5a)		Linear	$y = -192.49x + 19.62$	-192.49
14 (Fig. 5b)		Linear	$y = 1.3101x + 14.558$	1.3101
77 (Fig. 5c)		Linear	$y = -126.46x + 22.551$	-126.46
77 (Fig. 5d)		Polynomial order 2	$y = -30.574x^2 - 3.5648x + 18.311$	N/A
30°C				
14 (Fig. 6a)		Logarithmic	$y = -6.041\ln(x) - 6.4878$	N/A
14 (Fig. 6b)		Linear	$y = -2.7562x + 5.5156$	-2.7562
77 (Fig. 6c)		Linear	$y = 16.722x + 1.6253$	16.722
77 (Fig. 6d)		Polynomial order 2	y = -89.899x2 + 71.43x - 6.4874	N/A
<u>Mesophilic fungi (varying water activity)</u>				
Aspergillus sclerotiicarbonarius				

CBS 121853 (Fig. 7a)	Glycerol at 0.950_{aw}	Linear	y = 0.0907x + 1.589	0.0907
Aspergillus aculeatinus				
CBS 121872 (Fig. 7b)	Glycerol at 0.990_{aw}	Linear	y = -3.3533x + 1.5876	-3.3533
CBS 121872 (Fig. 7c)	Glycerol at 0.980_{aw}	Polynomial order 2	$y = 28.007x^2 - 17.053x + 3.5154$	N/A
CBS 121875 (Fig. 7d)	Glycerol at 0.980_{aw}	Linear	y = -3.8776x + 1.7822	-3.8776
Mesophilic fungi (varying temperature)				
Aspergillus sclerotiicarbonarius				
CBS 121853 (Fig. 3a)	Glycerol at 30°C	Linear	y = -0.3954x + 1.4405	-0.3954
Aspergillus aculeatinus				
CBS 121872 (Fig. 3b)	Glycerol at 35°C	Linear	y = -1.4903x + 1.3449	-1.4903
CBS 121872 (Fig. 3c)	Glycerol at 30°C	Polynomial order 2	$y = 7.0229x^2 - 9.6055x + 3.2171$	N/A
CBS 121875 (Fig. 3d)	Glycerol at 30°C	Linear	y = -3.9008x + 1.9369	-3.9008
Enzyme systems				
(from *Terfezia claveryi*)				
Lipoxygenase (Fig. 8a)	Hydroperoxy octadecadienoic acid (9-HPOD)	Linear	y = 1.6284x - 12.042	1.6284
Lipoxygenase (Fig. 8b)	Hydroperoxy octadecadienoic acid (13-HPOD)	Linear	y = 0.6408x - 4.8009	0.6408
(from *Agaricus bisporus*)				
Tyrosinase (Fig.8c)	Anisic acid	Linear	y = -4567.2x + 9.2446	-4567.2

[a]Seven types of trend-line were fitted (see *Experimental procedures*), curve which best-described the data and gave the highest r^2 value was selected.
[b]Where y and x denote values on y and x axes respectively.
[c]Based on y =mx+ c, where y is the y axis value, m is the gradient of the line, x is the x axis value and c is the y-intercept where the line crosses the y axis.

Table S7. Dehydration-rehydration treatments and culture conditions for assays of *S. cerevisiae* strains.

Strains and culture medium used for initial cultures	Details of dehydration treatment (if applicable)	Rehydration and assessment of subsequent growth
14 in YPDB	23°C by a convective method	Control with water<br>Control with xylitol<br>Rapid rehydration with water<br>Rapid rehydration with xylitol<br>Gradual rehydration with water<br>Gradual rehydration with xylitol
	30°C in a drying oven	Control with water<br>Control with xylitol<br>Rapid rehydration with water<br>Rapid rehydration with xylitol<br>Gradual rehydration with water<br>Gradual rehydration with xylitol<br>Rapid rehydration with xylitol and water<br>Gradual rehydration with xylitol and water
14 in SSNB	23°C by a convective method	Control with water<br>Control with xylitol<br>Rapid rehydration with water<br>Rapid rehydration with xylitol<br>Gradual rehydration with water<br>Gradual rehydration with xylitol
	30°C in a drying oven	Control with water<br>Control with xylitol<br>Rapid rehydration with water<br>Rapid rehydration with xylitol<br>Gradual rehydration with water<br>Gradual rehydration with xylitol<br>Rapid rehydration with xylitol and water<br>Gradual rehydration with xylitol and water
77 in YPDB	23°C by a convective method	Control with water<br>Control with xylitol<br>Rapid rehydration with water<br>Rapid rehydration with xylitol<br>Gradual rehydration with water<br>Gradual rehydration with xylitol
	30°C in a drying oven	Control with water<br>Control with xylitol<br>Rapid rehydration with water<br>Rapid rehydration with xylitol<br>Gradual rehydration with water<br>Gradual rehydration with xylitol<br>Rapid rehydration with xylitol and water<br>Gradual rehydration with xylitol and water

77 in SSNB	23°C by a convective method	Control with water<br>Control with xylitol<br>Rapid rehydration with water<br>Rapid rehydration with xylitol<br>Gradual rehydration with water<br>Gradual rehydration with xylitol
	30°C in a drying oven	Control with water<br>Control with xylitol<br>Rapid rehydration with water<br>Rapid rehydration with xylitol<br>Gradual rehydration with water<br>Gradual rehydration with xylitol<br>Rapid rehydration with xylitol and water<br>Gradual rehydration with xylitol and water
