# Supplementary material for: Microbial lag phase can be indicative of, or independent from, cellular stress
Source: Sci Rep. 2020 Apr 3;10:5948. doi: 10.1038/s41598-020-62552-4 (PMC7125082; doi:10.1038/s41598-020-62552-4)
Supplement: Supplementary file 2 — Supplementary Figures [file 41598_2020_62552_MOESM2_ESM.pdf]

Supplementary Information Figures

Microbial lag phase can be indicative of, or independent from, cellular stress

Philip G. Hamill[1], Andrew Stevenson[1], Phillip E. McMullan[1], James P. Williams[1], Abiann D. R. Lewis[1], Sudharsan S[2], Kath E. Stevenson[3], Keith D. Farnsworth[1], Galina Khroustalyova[4], Jon Y. Takemoto[5], John P. Quinn[1], Alexander Rapoport[4], and John E. Hallsworth[1]

[1]*Institute for Global Food Security, School of Biological Sciences, Queen's University Belfast, 19 Chlorine Gardens, Belfast, BT9 5DL, Northern Ireland.*
[2]*Department of Chemistry, PGP College of Arts and Science, NH-7, Karur Main Road, Paramathi, Namakkal, Tamil Nadu, 637 207, India*
[3]*Special Collections and Archives, McClay Library, Queen's University Belfast, 10 College Park Avenue, Belfast, BT7 1LP, Northern Ireland.*
[4]*Laboratory of Cell Biology, Institute of Microbiology and Biotechnology, University of Latvia, Jelgavas Str., 1-537, LV-1004 Riga, Latvia.*
[5]*Utah State University, Department of Biology, 5305 Old Main Hill Logan, UT 84322, USA.*

Supplementary Figures

Fig. S1. Progress of spore germination for four strains of xerophilic fungi: (**a**) *Aspergillus penicillioides* JH06THH; (**b**) *Xeromyces bisporus* FRR 3443; (**c**) *X. bisporus* FRR 1522; and (**d**) *Xerochrysium xerophilum* FRR 0530. Percentage germination was determined on malt-extract yeast-extract phosphate agar (MYPiA) supplemented with diverse stressor(s) and incubated at 30°C for up to 50 days. Media were supplemented with: glycerol (red lines), at 7.0 and 7.1 M, with water-activity values of 0.707 and 0.664, respectively; glycerol (5.5 M)+NaCl at 0.5, 1.0, 1.5 and 1.6 M (green lines), with water-activity values of 0.765, 0.741, 0.709 and 0.692, respectively; glycerol (5.5 M)+sucrose at 0.25, 0.50, 0.65 and 0.80 M (blue lines), with water-activity values of 0.734, 0.699, 0.674 and 0.637, respectively; glycerol (5.5 M) + glucose at 0.8 and 1.0 M + fructose at 0.80 and 1.0 M (yellow lines), with water activities of 0.694 and 0.649, respectively; and glycerol (5.5 M)+NaCl (0.5 M)+sucrose at 0.3 and 0.5 M (black lines), with water-activity values of 0.701 and 0.685, respectively (see Table S2). Two hundred spores, and 50 individual germinated spores were measured for germ-tube length, mean values are plotted, and grey bars indicate standard errors. Modified from Stevenson *et al*.[14] with permission from Wiley Online Library.

Fig. S2. Length of lag phase versus maximum rate of growth (optical density arbitrary units h^{-1}) for *Bacillus subtilis* in Belitzki minimal medium broth that had been supplemented with a range of stressors: (**a**) glycerol at 2.72 (♦), 2.26 (■), 1.81 (▲), 1.36 (×), 0.91 (-) and 0.45 M (●), and without glycerol (control; +); (**b**) proline at 2.61 (♦), 2.18 (■), 1.74 (▲), 1.30 (×), 0.87 (-), 0.43 M (●), and without proline (control; +); (**c**) glucose at 1.94 (♦), 1.62 (■), 1.30 (×), 0.97 M (-), 0.65 M (●), and 0.32 M (□); (**d**) $MgCl_2$ at 1.37 (♦), 1.14 (■), 0.91 (▲), 0.68 M (×), 0.46 (-), 0.23 nM (●), and without $MgCl_2$ (control; +); (**e**) sucrose at 1.31 (♦), 1.10 (■), 0.88 (▲), 0.66 (×), 0.44 (-), 0.22 M (●), and without sucrose (control; +); and (**f**) ammonium sulphate at 0.76 (♦), 0.51 (■), 0.25 M (▲), and without ammonium sulphate (control; +). All controls and treatments were carried out in triplicate. Data were obtained from Fig. S13 (see *Experimental procedures)*, and trend lines showing a strong correlation between data are solid and black (**b**; **c**; **d**; **e**; **f**), and a weak correlation is indicated by the dashed black line (**a**) (Table S5).

Fig. S3. Growth curves for *Saccharomyces cerevisiae* at 23°C: strain 14 in salt-supplemented nutrient broth (SSNB) (**a**) and yeast extract peptone dextrose broth (YPDB) (**b**); and strain 77 in SSNB (**c**) and YPDB (**d**). The water content of these cells was reduced to 8-10% w/w, and sterile distilled water or a 1 M xylitol solution (10 ml) was added to 50 mg of dried cells for determinations of cell viability (see *Expermental procedures*). Cells suspended in water (not subjectied to any dehydration treatment) (pink dots), and cells suspended in 1 M xylitol (no dehydration treatment) (orange dots), were used as controls. For dehydrated cells, rehydration treatments were either rapid, using water (grey dots); rapid, using 1 M xylitol (brown dots); gradual, using water vapour then rapid in water (purple dots); or gradual rehydration with water vapor, then rapid in 1 M xylitol (turquoise dots). Mean values are plotted, and grey bars indicate standard errors.

Fig. S4. Growth curves for *Saccharomyces cerevisiae* at 30°C: strain 14 in salt-supplemented nutrient broth (SSNB) (**a**) and yeast extract peptone dextrose broth (YPDB) (**b**); and strain 77 in SSNB (**c**) and YPDB (**d**). The water content of these cells was reduced to 8-10% w/w and for determinations of cell viability, sterile distilled water or a 1 M xylitol solution (10 ml) was added to 50 mg of dried cells (see *Expermental procedures*). Cells suspended in water (not subjectied to any dehydration treatment) (pink dots), and cells suspended in 1 M xylitol (no dehydration treatment) (orange dots), were used as controls. For dehydrated cells, rehydration treatments were either rapid, using water (grey dots); rapid, using 1 M xylitol (brown dots); gradual, using water vapour then rapid in water (purple dots); or gradual rehydration with water vapor, then rapid in 1 M xylitol (turquoise dots) or for xylitol dehydrated cells; gradual, using water vapour then rapid in water (×); and gradual, using water vapour then rapid in 1 M xylitol (▲). All conditions were assessed in triplicate, mean values were plotted, and grey bars indicate standard error.

Fig. S5. Growth curves for *Mrakia frigida* (DSM 70883) on malt-extract yeast-extract phosphate agar (MYPiA) at 1.7°C, supplemented with a range of stressors: (**a**) sucrose (1.5 M); (**b**) glucose (2.8 M); (**c**) glycerol (4.3 M); (**d**) glycerol (2.7 M); (**e**) NaCl (2.5 M); and (**f**) $MgCl_2$ (1 M) and combined solute-treatment 2 (20% glycerol, 10% sucrose, 1% KCl plus 5% NaCl [w/v]). For each treatment, there were six replicates; these are

shown in red, blue, orange, green, purple, and turquoise on each plot.

Fig. S6. Maximum growth rate for *Escherichia coli* BL21 (syn. DE3) versus stressor concentration in Luria–Bertani (LB) broth (23°C) supplemented with: (**a**) ethanol; (**b**) butanol; and (**c**) urea. The last three datapoints of each curve (corresponding to the three highest stressor conncentrations) were used to determine the theoretical maximum concentration that would permit growth for each stressor, using a linear trend line (dotted line) for (**d**) ethanol; (**e**) butanol; and (**f**) urea.

Fig. S7. Growth curves for *Bacillus subtilis* 168 in Belitzki minimal medium broth, supplemented with a range of stressors: (**a**) betaine at 2.56 (♦), 2.13 (■), 1.71 (▲), 1.28 (×), 0.85 (-) and 0.43 M(●), and without betaine (control; +); (**b**) guanidine hydrochloride at 150 (♦), 125 (■), 100 (▲), 75 (×), 50 (-) and 25 nM (●), and without guanidine hydrochloride (control; +); (**c**) ammonium sulphate at 0.76 (♦), 0.51 (■), 0.25 M (▲), and without ammonium sulphate (control; +); and (**d**) polyethylene glycol 600 at 400 (♦), 333 (■), 267 (▲), 200 (×), 133 (-) and 67 nM (●), and without polyethylene glycol 600 (control; +). All conditions were assessed in triplicate, mean values are plotted, and grey bars indicate standard errors.

Fig. S8. Length of lag phase versus maximum rate of germination for eight strains of extremophilic fungi: (**a**) *Aspergillus penicillioides* JH06GBM; (**b**) *A. penicillioides* JH06THJ; (**c**) *Eurotium amstelodami* FRR 2792; (d) *Eurotium echinulatum* FRR 5040; (e) *Eurotium halophilicum* FRR 2471; (**f**) *Eurotium repens* JH06JPD; (**g**) *Xeromyces bisporus* FRR 0025; and (**h**) *X. bisporus* FRR 2347. Germination was assessed on malt-extract yeast-extract phosphate agar (MYPiA) supplemented with diverse stressor(s) and incubated at 30ºC for up to 50 days. Media were supplemented with: glycerol (red dots), at 7.0 and 7.1 M, with water-activity values of 0.707 and 0.664, respectively; glycerol (5.5 M)+NaCl at 0.5, 1.0, 1.5, and 1.6 M (green dots), with water-activity values of 0.765, 0.741, 0.709 and 0.692, respectively; glycerol (5.5 M)+sucrose at 0.25, 0.50, 0.65, and 0.80 M (blue dots), with water-activity values of 0.734, 0.699, 0.674, and 0.637, respectively; glycerol (5.5 M) + glucose at 0.8 and 1.0 M + fructose at 0.80 and 1.0 M (yellow lines), with water activities of 0.694 and 0.649, respectively; glycerol (5.5 M)+NaCl (0.5 M)+sucrose at 0.3 and 0.5 M (black dots), with water-activity values of 0.701 and 0.685, respectively. Data were obtained from Fig. S13

(see *Experimental procedures)*, and trend lines showing a strong correlation and moderate correlation between data are solid and black (**a**; **c**; **e**; **f**; **h**), a weak correlation is indicated by a dashed black line (**b**), while dashed grey lines indicate inconsistency between lag and growth rates (i.e. no correlation was observed) (**d**; **g**) (Table S5).

Fig. S9. Length of lag phase versus maximum rate of growth *Mrakia frigida* (DSM 70883) on malt-extract, yeast-extract phosphate agar (MYPiA) at 1.7°C, supplemented with a range of stressors: (**a**) sucrose (1.5 M); (**b**) glucose (2.8 M); (**c**) glycerol (4.3 M); (**d**) glycerol (2.7 M); (**e**) NaCl (2.5 M); and (**f**) $MgCl_2$ (1 M) and combined solute-treatment 2 (20% glycerol, 10% sucrose, 1% KCl plus 5% NaCl [w/v]). Trend lines showing strong correlations between data are solid and black (**a**; **d** and **e**), while moderate correlations are indicated by a dashed black line (**b**; **c**; and **f**) (Table S5).

Fig. S10. Length of lag phase versus maximum rate of growth for *Escherichia coli* BL21 (syn. DE3) in Luria–Bertani (LB) broth, supplemented with a range of stressors: (**a**) ethanol at 600 (orange ×), 650 (grey ×), 700 (yellow ×), 750 (blue ×), 800 (green ×) and 850 nM (black ×); (**b**) butanol at 70 (purple ×), 80 (orange ×), 90 (grey ×), 100 (yellow ×), 110 (blue ×) and 120 nM (green ×); and (**c**) urea at 295 (purple ×), 389 (orange ×), 482 (grey ×), 574 (yellow ×), 663 (blue ×), 751 (grey ×), and 838 nM (black ×). Data were obtained from Fig. S14 (see *Experimental procedures)*, and trend lines showing a strong correlation between data are solid and black (**b** and **c**), while a moderate correlation is indicated by a dashed black line (**a**) (Table S5).

Fig. S11. Growth curves for *Bacillus subtilis* 168 in Belitzki minimal medium broth, supplemented with a range of stressors: (**a**) glycerol at 2.72 (♦), 2.26 (■), 1.81 (▲), 1.36 (×), 0.91 (-) and 0.45 M (●), and without glycerol (control; +); (**b**) proline at 2.61 (♦), 2.18 (■), 1.74 (▲), 1.30 (×), 0.87 (-), 0.43 M (●), and without proline (control; +); (**c**) glucose at 1.94 (♦), 1.62 (■), 1.30 (×), 0.97 M (-), 0.65 M (●), and 0.32 M (□); (**d**) $MgCl_2$ at 1.37 (♦), 1.14 (■), 0.91 (▲), 0.68 M (×), 0.46 (-), 0.23 nM (●), and without $MgCl_2$ (control; +); (**e**) sucrose at 1.31 (♦), 1.10 (■), 0.88 (▲), 0.66 (×), 0.44 (-), 0.22 M (●), and without sucrose (control; +); and (**f**) polyethylene glycol 6000 at 50 (♦), 42 (■), 33 (▲), 25 (×), 17 (-) and 8 nM (●) and without polyethylene glycol 6000 (control; +).

Fig. S12. Length of lag phase versus specific growth rate for: *Pseudomonas fluorescens* (ATCC 17400) (**a**, **f** and **k**), *Salmonella typhlmurium* (ATCC 13311) (**d**, **i** and **n**), *Staphylococcus aureus* (ATCC 13566) in nutrient broth (**e**, **j** and **o**), *Brochothrix thermosphacta* (ATCC 12706) (**b**, **g**, and **l**), and *Enterococcus faecalis* (ATCC 7080) in brain heart infusion broth (**c**, **h** and **m**). In each case, media were supplemented with glycerol, NaCl, or sucrose (Li and Torres, 1993): (**a**) *P. fluorescens* with NaCl to a range of water activities: 0.980 at 4°C (♦), 0.982 at 8°C (ж),0.956 at 8°C (X), 0.981 at 16°C (●),0.956 at 16°C (■), 0.981 at 26°C (-) and 0.955 at 26°C (▲); (**b**) *B. thermosphacta* with NaCl to a range of water activities: 0.968 at 4°C (▲), 0.952 at 4°C (×); 0.968 at 8°C (♦), 0.952 at 8°C (■), 0.968 at 16°C (-), 0.952 at 16°C (ж), 0.967 at 26°C (+), and 0.952 at 26°C (●); (**c**) *E. faecalis* with NaCl to a range of water activities: 0.978 at 12°C (▲), 0.982 at 16°C (♦), 0.958 at 16°C (×), 0.981 at 20°C (●), 0.956 at 20°C (■), and 0.953 at 26°C (ж); (**d**) *S. typhimurium* with NaCl to a range of water activities: 0.983 at 12°C (■), 0.967 at 12°C (×), 0.984 at 16°C (ж), 0.965 at 16°C (▲), 0.980 at 20°C (●),0.965 at 20°C (♦),0.984 at 26°C (+) and 0.964 at 26°C (-); (**e**) *S. aureus* with NaCl to a range of water activities: 0.968 at 12°C (■),0.950 at 12°C (×), 0.967 at 16°C (♦), 0.950 at 16°C (▲), 0.968 at 20°C (●), 0.950 at 20°C (ж), 0.950 at 26°C (-), and 0.970 at 26°C (+); (**f**) *P. fluorescens* with glycerol to a range of water activities: 0.978 at 4°C (■), 0.956 at 4°C (×), 0.980 at 8°C (♦),0.956 at 8°C (▲), 0.981at 16°C (-), 0.956 at 16°C (ж), 0.979 at 26°C (+), and 0.955 at 26°C (●); (**g**) *B. thermosphacta* with glycerol to a range of water activities: 0.972 at 4°C (■), 0.950 at 4°C (×), 0.972 at 8°C (♦), 0.950 at 8°C (▲), 0.972 at 16°C (●), 0.949 at 16°C (ж) 0.973 at 26°C (+) and 0.948 at 26°C (-); (**h**) *E. faecalis* with glycerol to a range of water activities: 0.968 at 12°C (♦),0.946 at 12°C (×), 0.968 at 16°C (ж), 0.946 at 16°C (■), 0.968 at 20°C (-), 0.944 at 20°C (▲) and 0.944 at 26°C (●); (**i**) *S. typhimurium* with glycerol to a range of water activities: 0.982 at 12°C (▲), 0.968 at 12°C (×), 0.983 at 16°C (♦), 0.964 at 16°C (■), 0.983 at 20°C (●), 0.969 at 20°C (ж), 0.969 at 26°C (-) and 0.982 at 26°C (+); (**j**) *S. aureus* with glycerol to a range of water activities: 0.977 at 12°C (■), 0.975 at 16°C (▲), 0.955 at 16°C (×), 0.974 at 20°C (ж), 0.950 at 20°C (♦), 0.970 at 26°C (-), and 0.952 at 26°C (●); (**k**) *P. fluorescens* with sucrose to a range of water activities: 0.980 at 4°C (×), 0.980 at 8°C (■), 0.980 at 16°C (ж), 0.960 at 16°C (▲), 0.978 at 26°C (●), and 0.955 at 26°C (♦); (**l**) *B. thermosphacta* with sucrose to a range of water activities: 0.969 at 4°C (×), 0.969 at 8°C (ж), 0.958 at 8°C (■),0.972 at 16°C (●), 0.957 at 16°C (▲), 0.972 at 26°C (-) and 0.951 at 26°C (♦); (**m**) *E. faecalis*

with sucrose to a range of water activities: 0.979 at 12°C (×), 0.978 at 16°C (♦),0.980 at 20°C (ж), 0.954 at 20°C (■), 0.981 at 26°C (●) and 0.950 at 26°C (▲); (**n**) *S. typhimurium* with sucrose to a range of water activities: 0.974 at 12°C (▲), 0.967 at 12°C (X), 0.978 at 16°C (ж), 0.967 at 16°C (■), 0.977 at 20°C (●),0.964 at 20°C (♦),0.975 at 26°C (+) and 0.964 at 26°C (-); (**o**) *S. aureus* with sucrose to a range of water activities: 0.980 at 12°C (■),0.977 at 16°C (▲), 0.953 at 16°C (×), 0.978 at 20°C (●), 0.950 at 20°C (♦), 0.980 at 26°C (-) and 0.947 at 26°C (ж). Trend lines showing a strong correlation between data are solid and black (**a**-**g**; **i**-**k** and **n**-**o**) while moderate correlations are indicated by a dashed black line (**h; l** and **m**) (Table S5).

Fig. S13. Progress of spore germination for eight strains of extremophilic fungi: (**a**) *A. penicillioides* JH06GBM; (**b**) *A. penicillioides* JH06THJ; (**c**) *Eurotium amstelodami* FRR 2792; (**d**) *E. echinulatum* FRR 5040; (**e**) *E. halophilicum* FRR 2471; (**f**) *E. repens* JH06JPD; (**g**) *X. bisporus* FRR 0025; and (h) *X. bisporus* FRR 2347. Germination was assessed on malt-extract yeast-extract phosphate agar (MYPiA) supplemented with diverse stressor(s) and incubated at 30ºC for up to 50 days. Media were supplemented with: glycerol (red lines), at 7.0 and 7.1 M, with water-activity values of 0.707 and 0.664, respectively; glycerol (5.5 M) + NaCl at 0.5, 1.0, 1.5 and 1.6 M (green lines), with water-activity values of 0.765, 0.741, 0.709 and 0.692, respectively; glycerol (5.5 M) + sucrose at 0.25, 0.50, 0.65 and 0.80 M (blue lines), with water-activity values of 0.734, 0.699, 0.674 and 0.637, respectively; glycerol (5.5 M) + glucose at 0.8 and 1.0 M + fructose at 0.80 and 1.0 M (yellow lines), with water activities of 0.694 and 0.649, respectively; and glycerol (5.5 M) + NaCl (0.5 M) + sucrose at 0.3 and 0.5 M (black lines), with water-activity values of 0.701 and 0.685, respectively (Table S2). Two hundred spores, and 50 spores that had germinated (and were spatially separate from others), were used to determine mean germ-tube length: these mean values were plotted, and grey bars indicate standard errors. Modified from Stevenson *et al*.[14], with permission from Wiley Online Library.

Fig. S14. Growth curves for *Escherichia coli* BL21 (syn. DE3) in Luria–Bertani (LB) broth, supplemented with a range of stressors: (**a**) ethanol at 600 (orange line), 650 (grey line), 700 (yellow line), 750 (blue line), 800 (green line) and 850 nM (black line); (**b**) butanol at 70 (purple line), 80 (orange line), 90 (grey line), 100 (yellow line), 110 (blue line) and 120 nM (green line); (**c**) urea at 295 (purple line), 389 (orange line),

482 (grey line), 574 (yellow line), 663 (blue line), 751 (grey line), and 838 nM (black line).

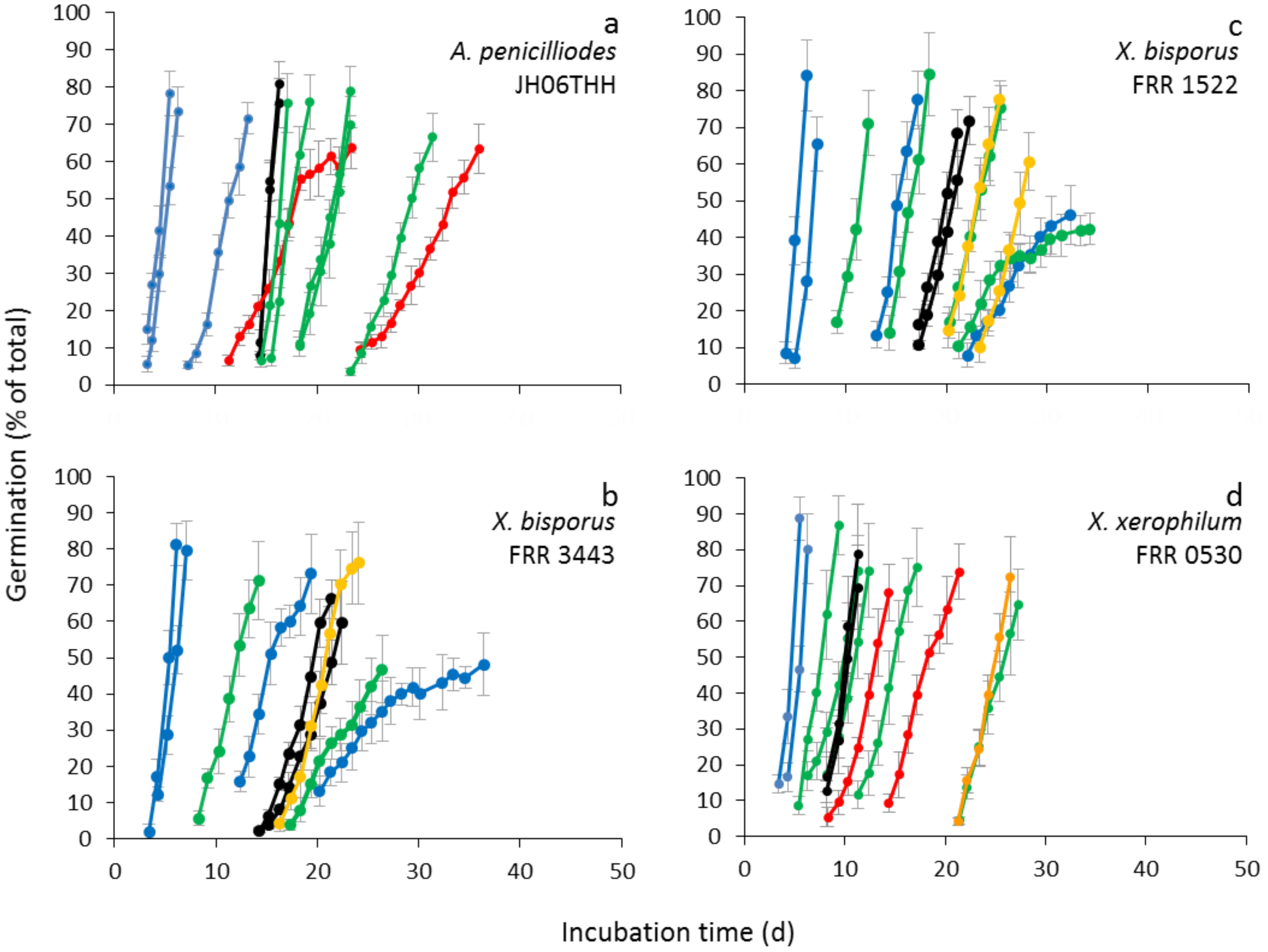
a
A. penicilliodes
JH06THH
c
X. bisporus
FRR 1522
b
X. bisporus
FRR 3443
d
X. xerophilum
FRR 0530
Germination (% of total)
Incubation time (d)
0
10
20
30
40
50
60
70
80
90
100

Figure. S1

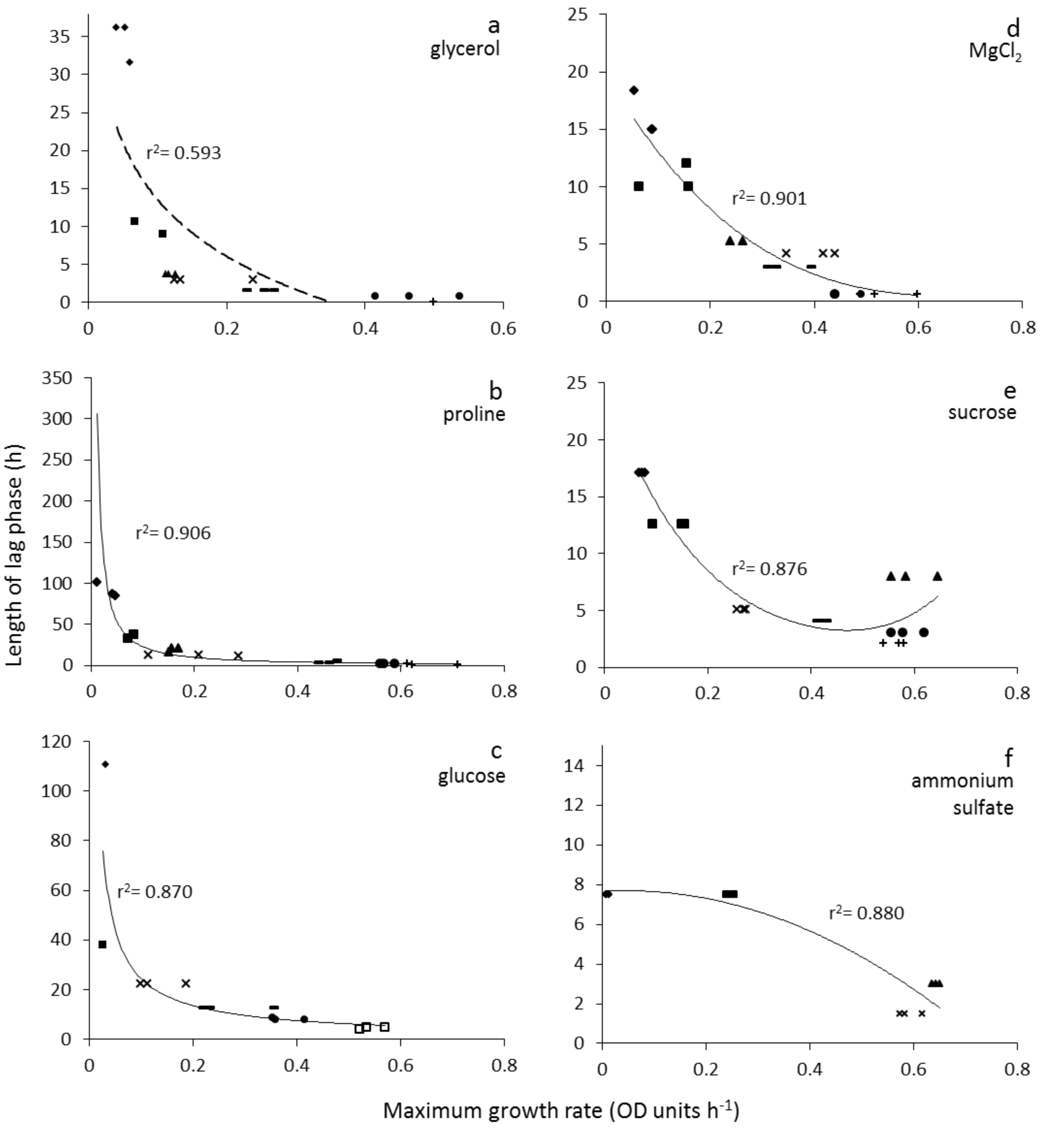
a
glycerol
r2= 0.593
b
proline
r2= 0.906
c
glucose
r2= 0.870
d
MgCl2
r2= 0.901
e
sucrose
r2= 0.876
f
ammonium
sulfate
r2= 0.880
Length of lag phase (h)
Maximum growth rate (OD units h-1)

Figure. S2

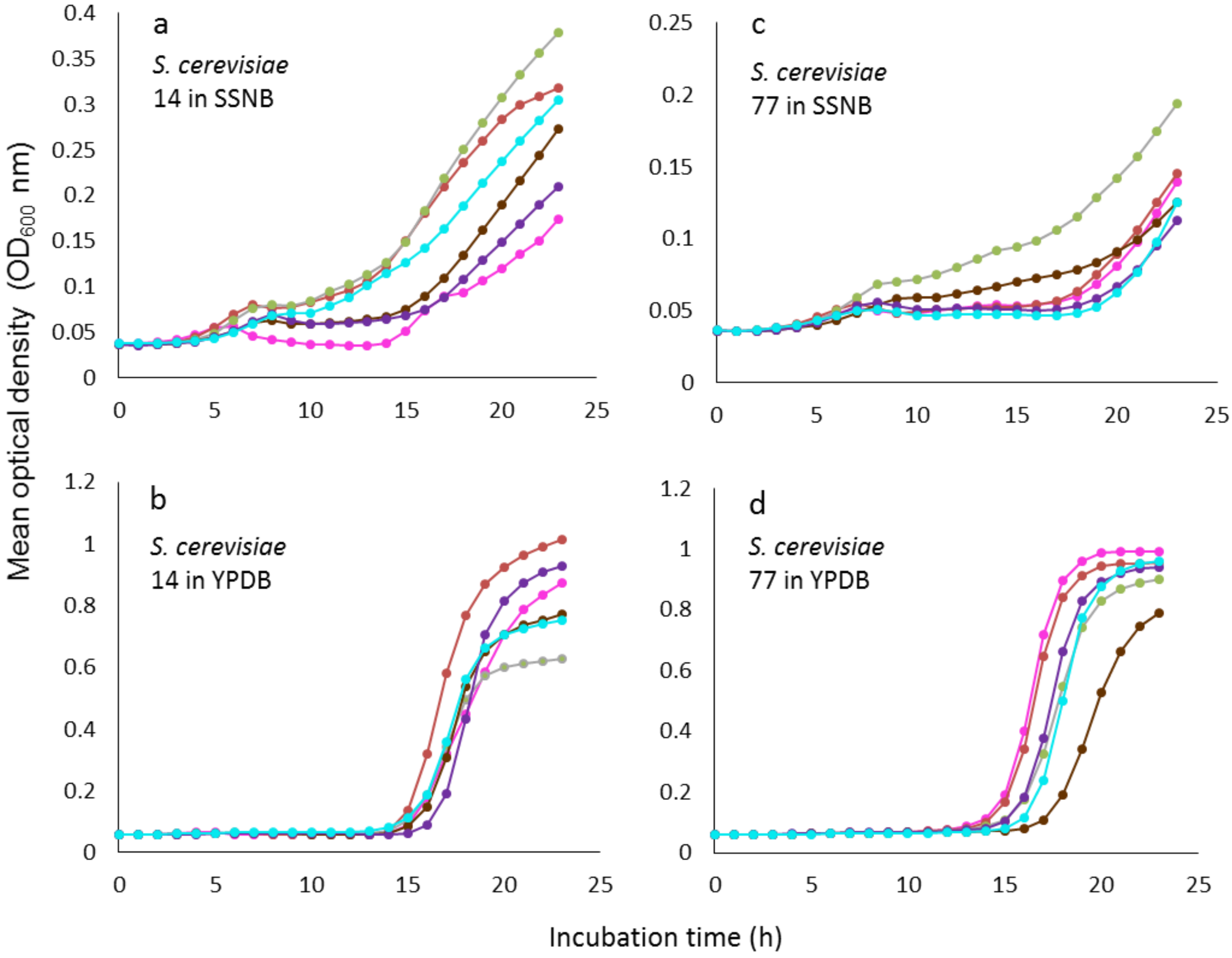

Mean optical density (OD600 nm)
a
S. cerevisiae
14 in SSNB
b
S. cerevisiae
14 in YPDB
c
S. cerevisiae
77 in SSNB
d
S. cerevisiae
77 in YPDB
Incubation time (h)

Figure. S3

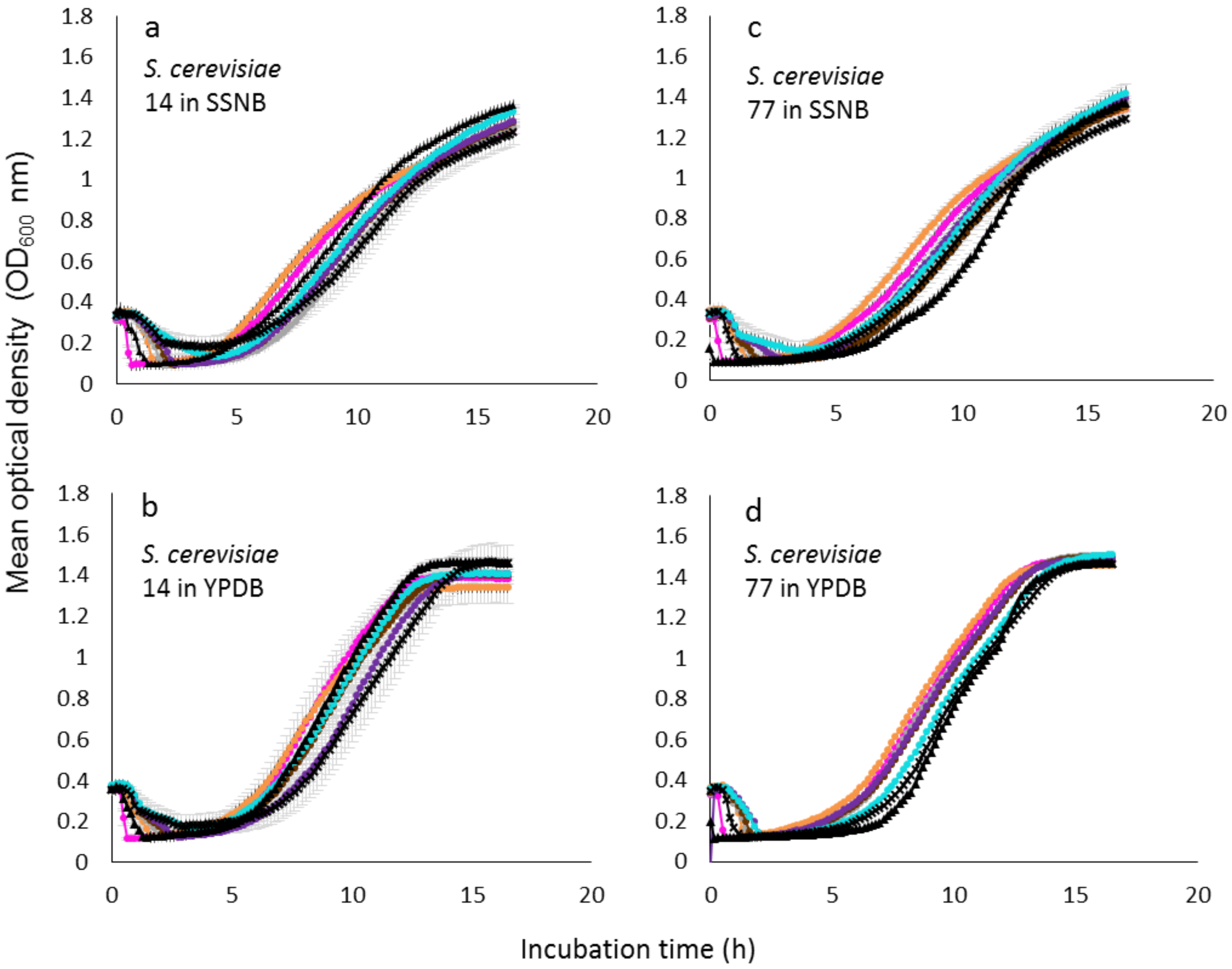
a
S. cerevisiae
14 in SSNB
b
S. cerevisiae
14 in YPDB
c
S. cerevisiae
77 in SSNB
d
S. cerevisiae
77 in YPDB
Mean optical density (OD600 nm)
Incubation time (h)
0
0.2
0.4
0.6
0.8
1
1.2
1.4
1.6
1.8
0
5
10
15
20

Figure. S4

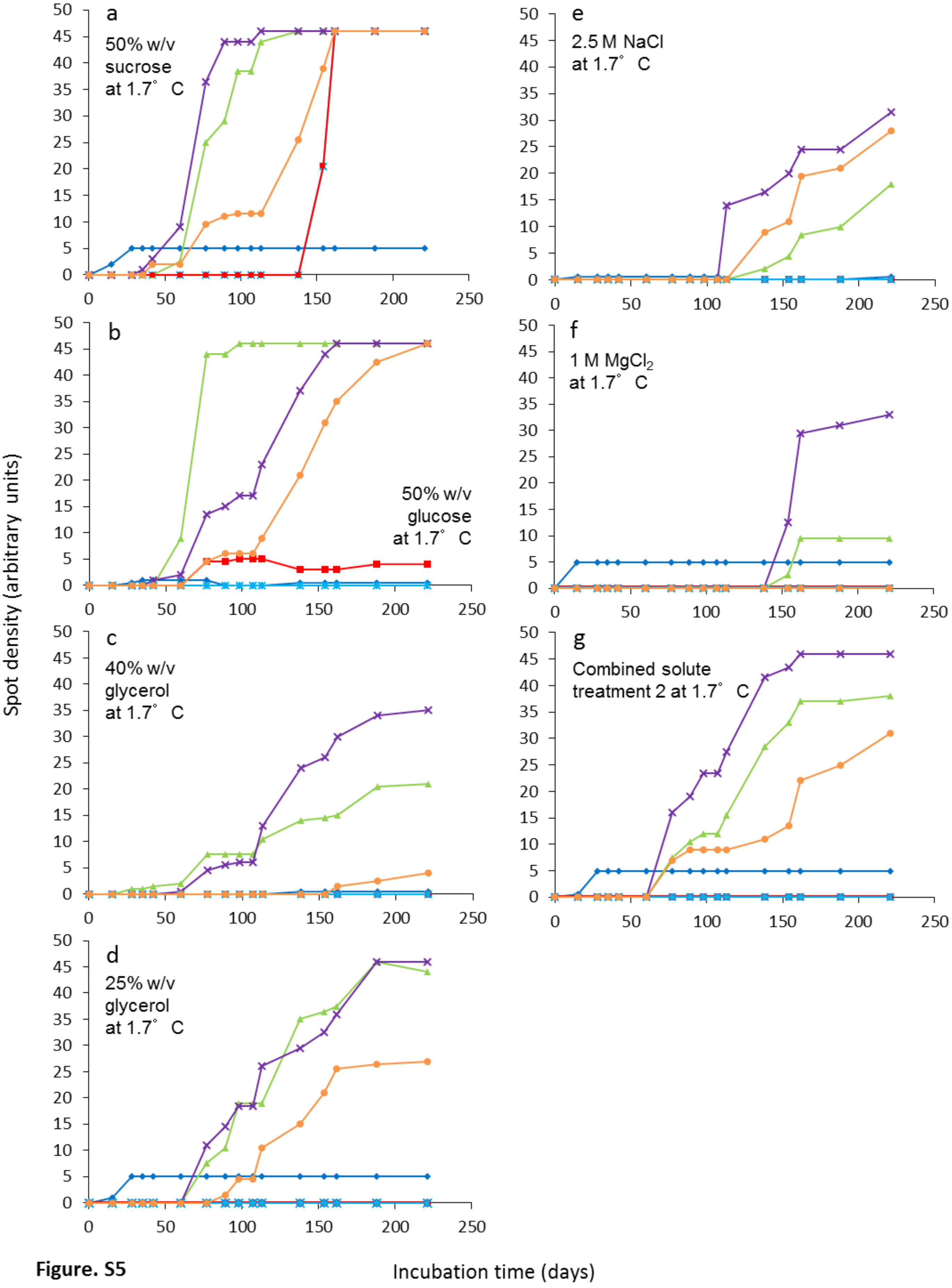

a
50% w/v sucrose at 1.7° C
b
50% w/v glucose at 1.7° C
c
40% w/v glycerol at 1.7° C
d
25% w/v glycerol at 1.7° C
e
2.5 M NaCl at 1.7° C
f
1 M $MgCl_2$ at 1.7° C
g
Combined solute treatment 2 at 1.7° C
Spot density (arbitrary units)
Incubation time (days)

Figure. S5

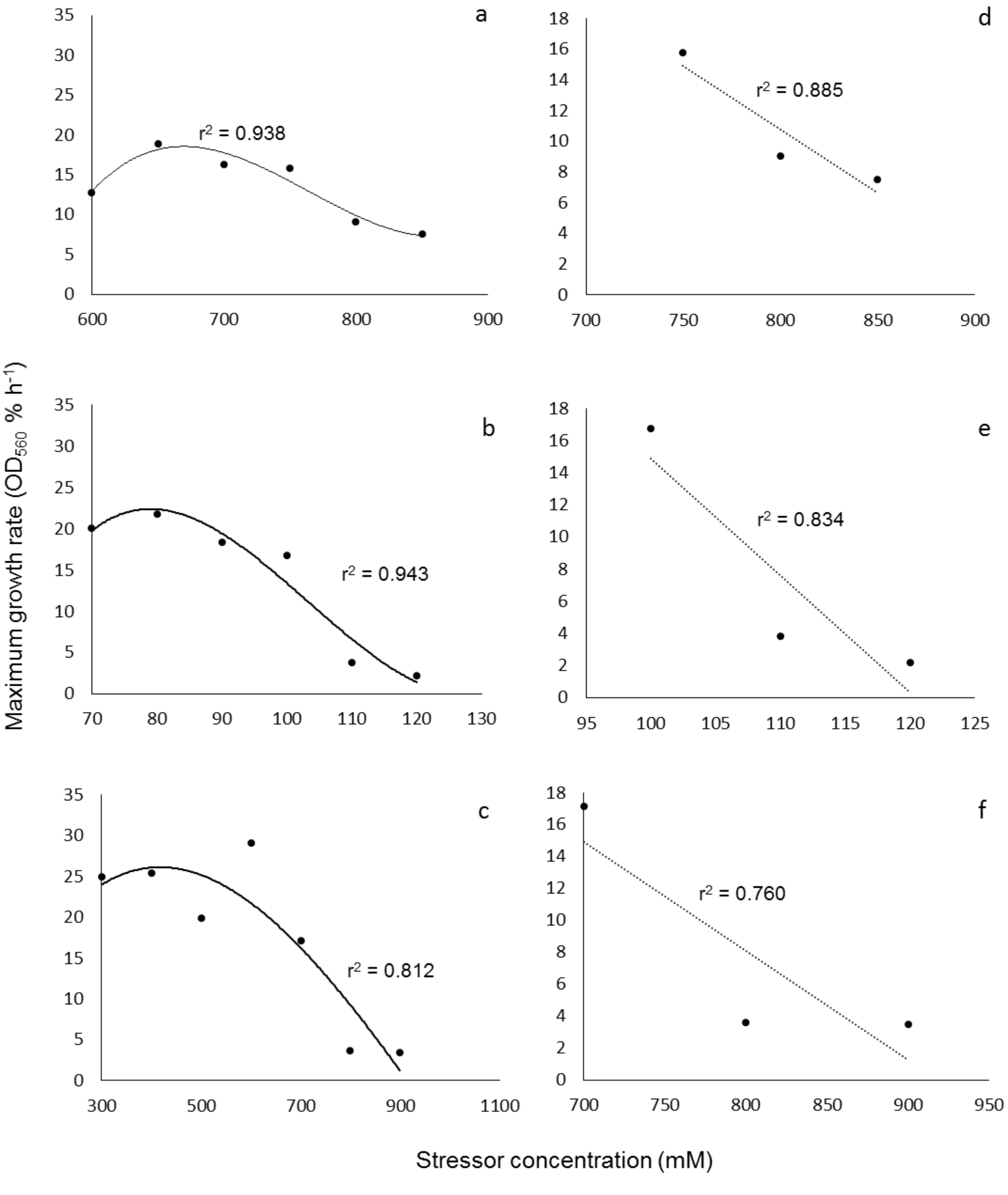
a
r2 = 0.938
b
r2 = 0.943
c
r2 = 0.812
d
r2 = 0.885
e
r2 = 0.834
f
r2 = 0.760
Maximum growth rate (OD560 % h-1)
Stressor concentration (mM)

Figure. S6

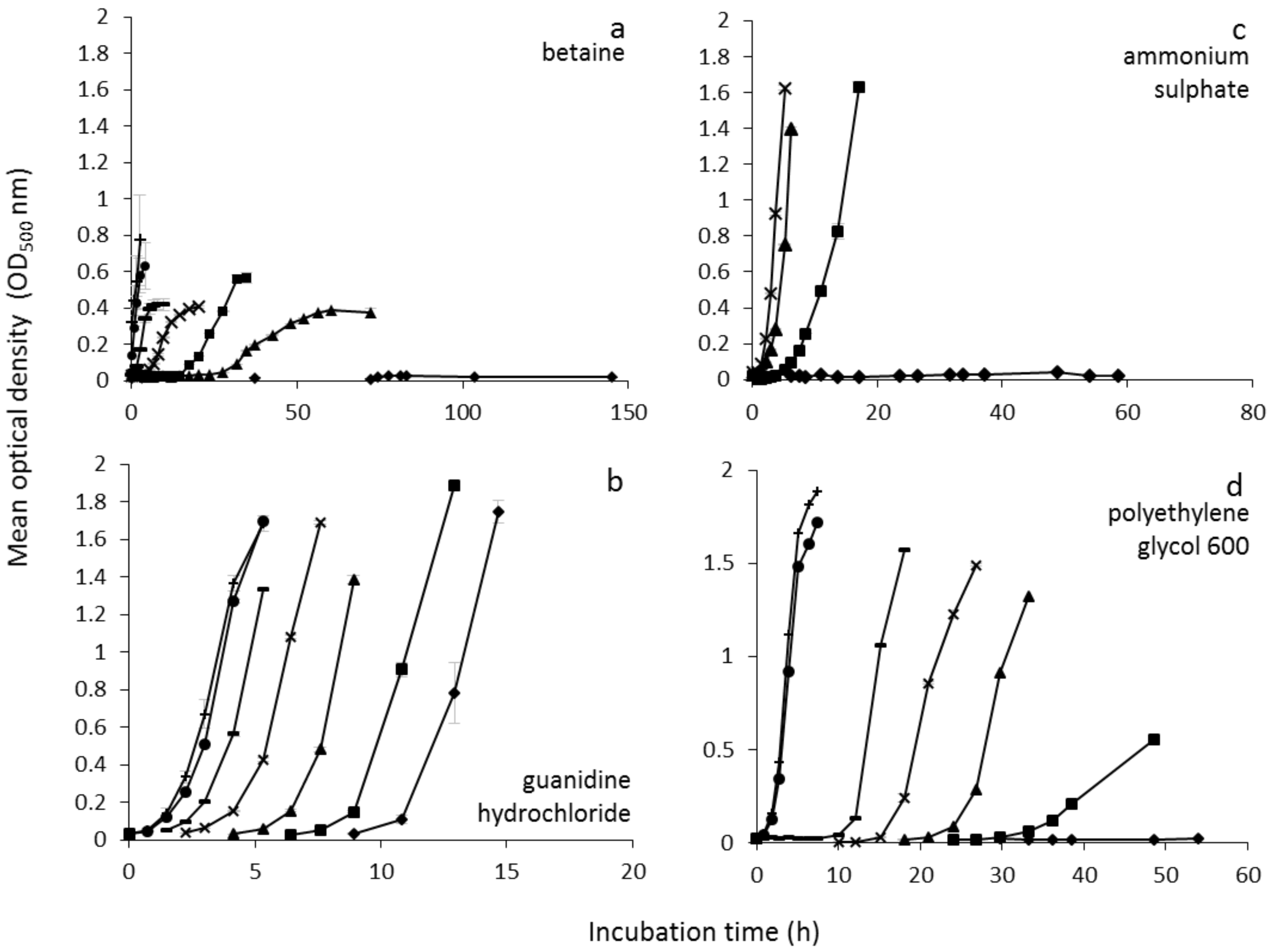
a
betaine
b
guanidine hydrochloride
c
ammonium sulphate
d
polyethylene glycol 600
Mean optical density (OD$_{500}$ nm)
Incubation time (h)

Figure. S7

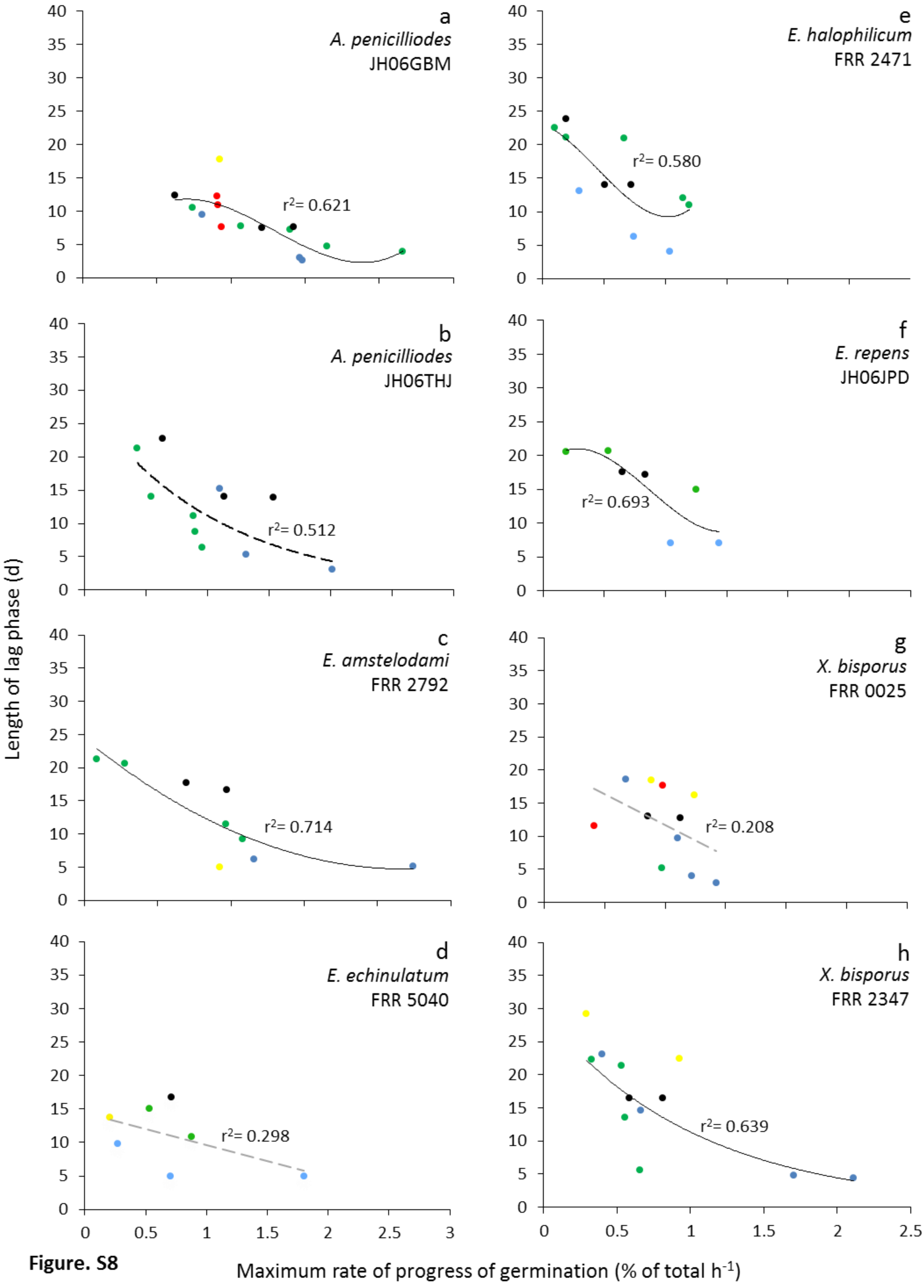
a
A. penicilliodes
JH06GBM
r²= 0.621
e
E. halophilicum
FRR 2471
r²= 0.580
b
A. penicilliodes
JH06THJ
r²= 0.512
f
E. repens
JH06JPD
r²= 0.693
c
E. amstelodami
FRR 2792
r²= 0.714
g
X. bisporus
FRR 0025
r²= 0.208
d
E. echinulatum
FRR 5040
r²= 0.298
h
X. bisporus
FRR 2347
r²= 0.639
Length of lag phase (d)
Maximum rate of progress of germination (% of total h-1)

Figure. S8

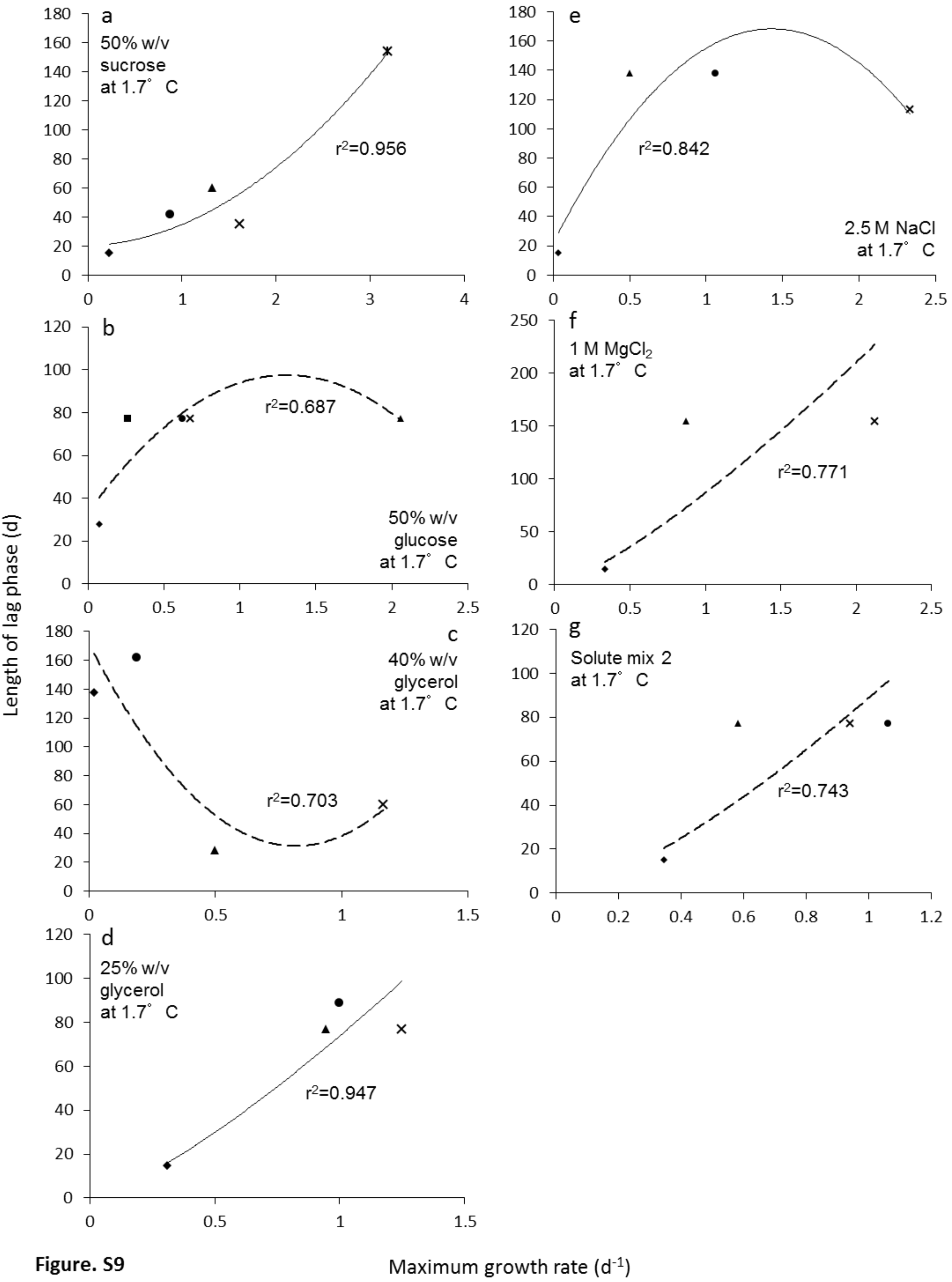
a
50% w/v sucrose at 1.7° C
r2=0.956
b
50% w/v glucose at 1.7° C
r2=0.687
c
40% w/v glycerol at 1.7° C
r2=0.703
d
25% w/v glycerol at 1.7° C
r2=0.947
e
2.5 M NaCl at 1.7° C
r2=0.842
f
1 M MgCl2 at 1.7° C
r2=0.771
g
Solute mix 2 at 1.7° C
r2=0.743
Length of lag phase (d)
Maximum growth rate (d-1)

Figure. S9

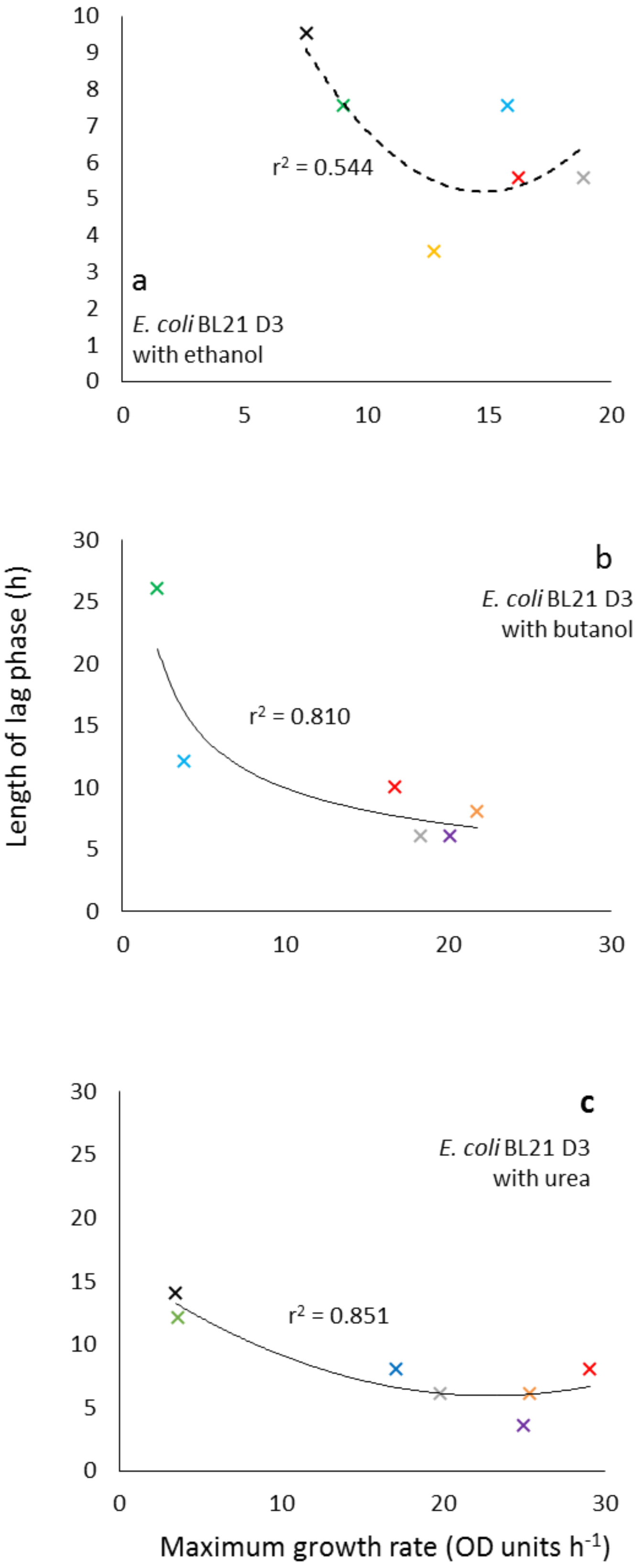
a
E. coli BL21 D3
with ethanol
r² = 0.544
b
E. coli BL21 D3
with butanol
r² = 0.810
c
E. coli BL21 D3
with urea
r² = 0.851
Length of lag phase (h)
Maximum growth rate (OD units h⁻¹)

Figure. S10

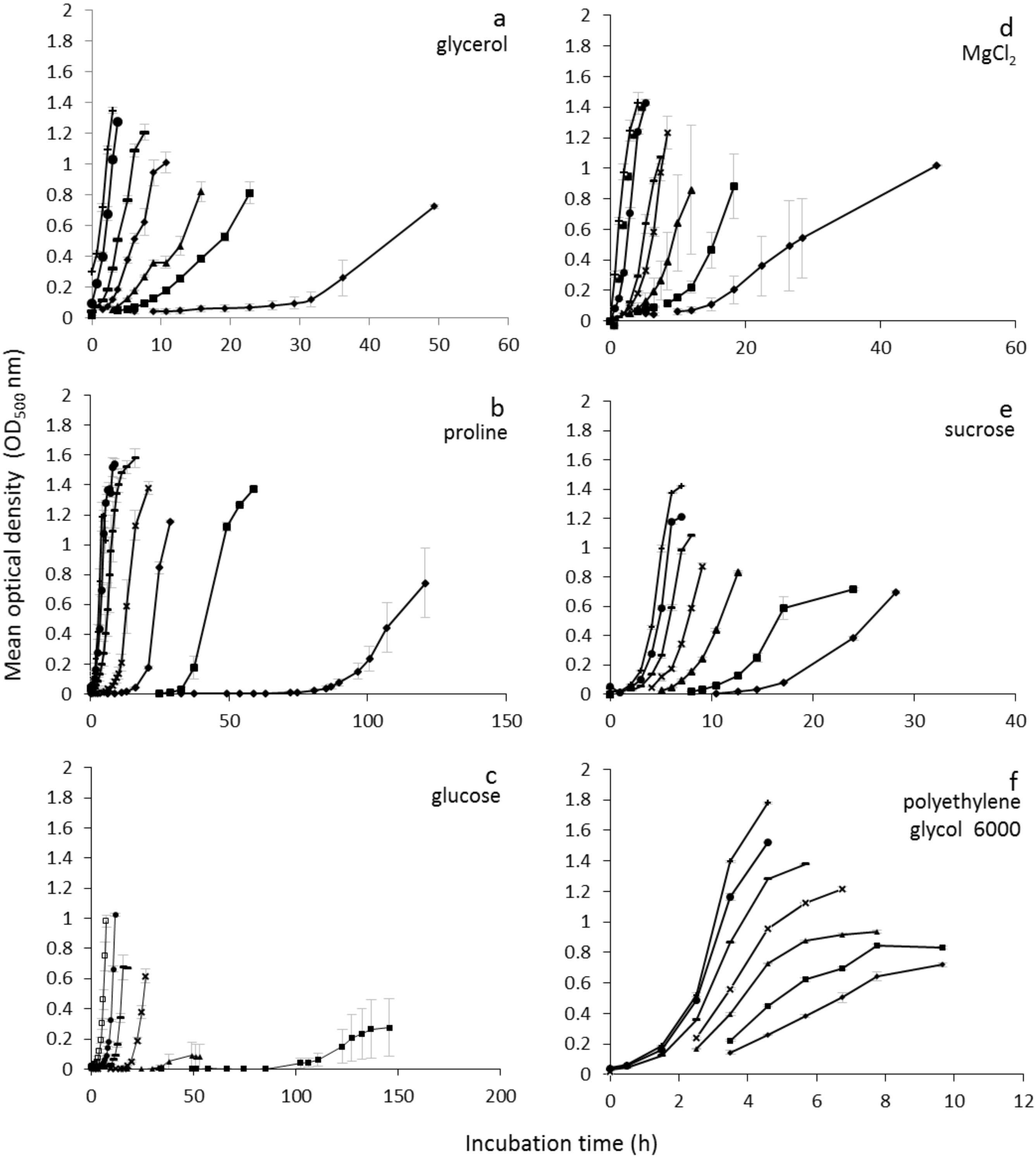

a
glycerol
b
proline
c
glucose
d
$MgCl_2$
e
sucrose
f
polyethylene glycol 6000
Mean optical density (OD_{500} nm)
Incubation time (h)

Figure. S11

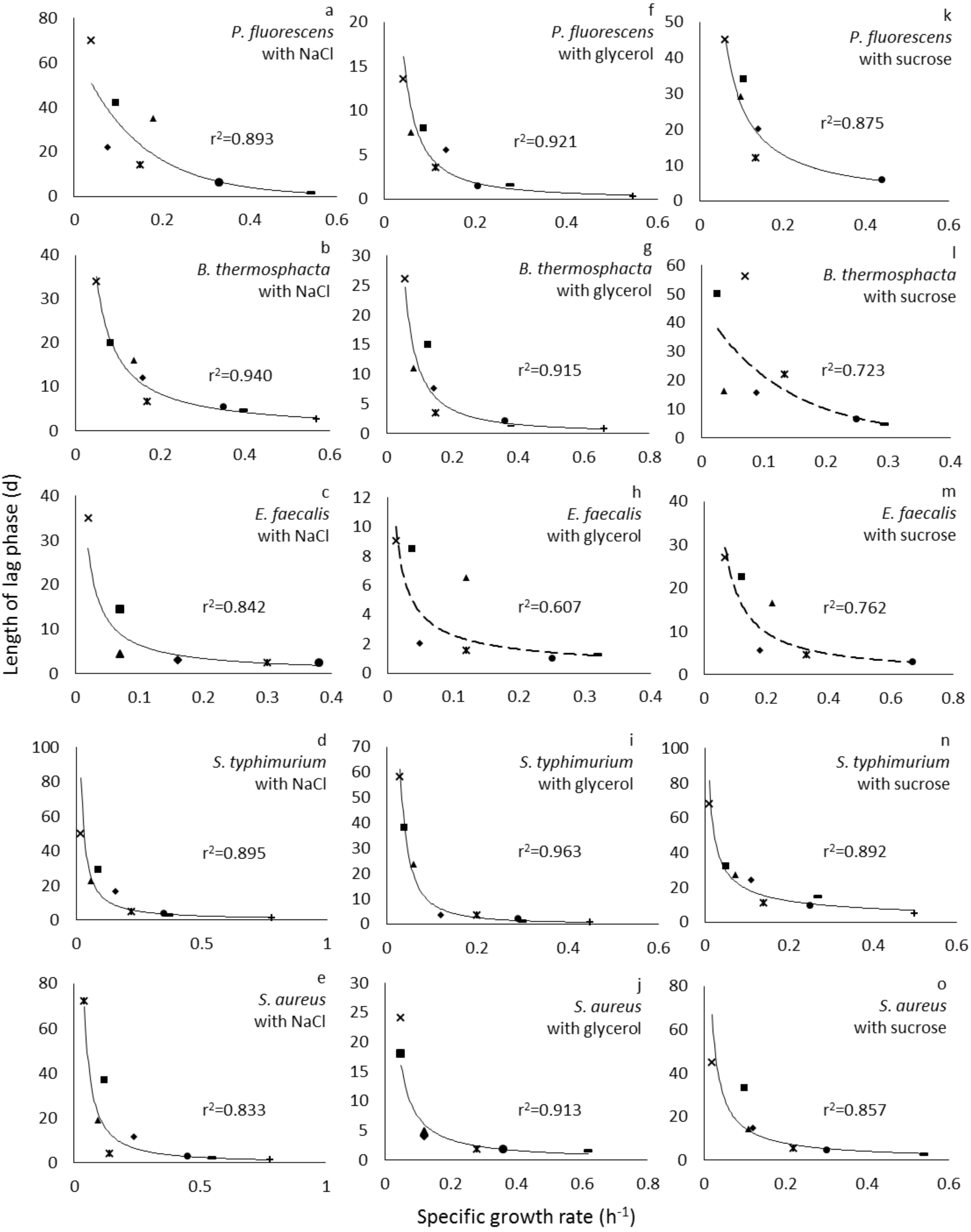
a
P. fluorescens
with NaCl
r2=0.893
f
P. fluorescens
with glycerol
r2=0.921
k
P. fluorescens
with sucrose
r2=0.875
b
B. thermosphacta
with NaCl
r2=0.940
g
B. thermosphacta
with glycerol
r2=0.915
l
B. thermosphacta
with sucrose
r2=0.723
c
E. faecalis
with NaCl
r2=0.842
h
E. faecalis
with glycerol
r2=0.607
m
E. faecalis
with sucrose
r2=0.762
d
S. typhimurium
with NaCl
r2=0.895
i
S. typhimurium
with glycerol
r2=0.963
n
S. typhimurium
with sucrose
r2=0.892
e
S. aureus
with NaCl
r2=0.833
j
S. aureus
with glycerol
r2=0.913
o
S. aureus
with sucrose
r2=0.857
Length of lag phase (d)
Specific growth rate (h-1)

Figure. S12

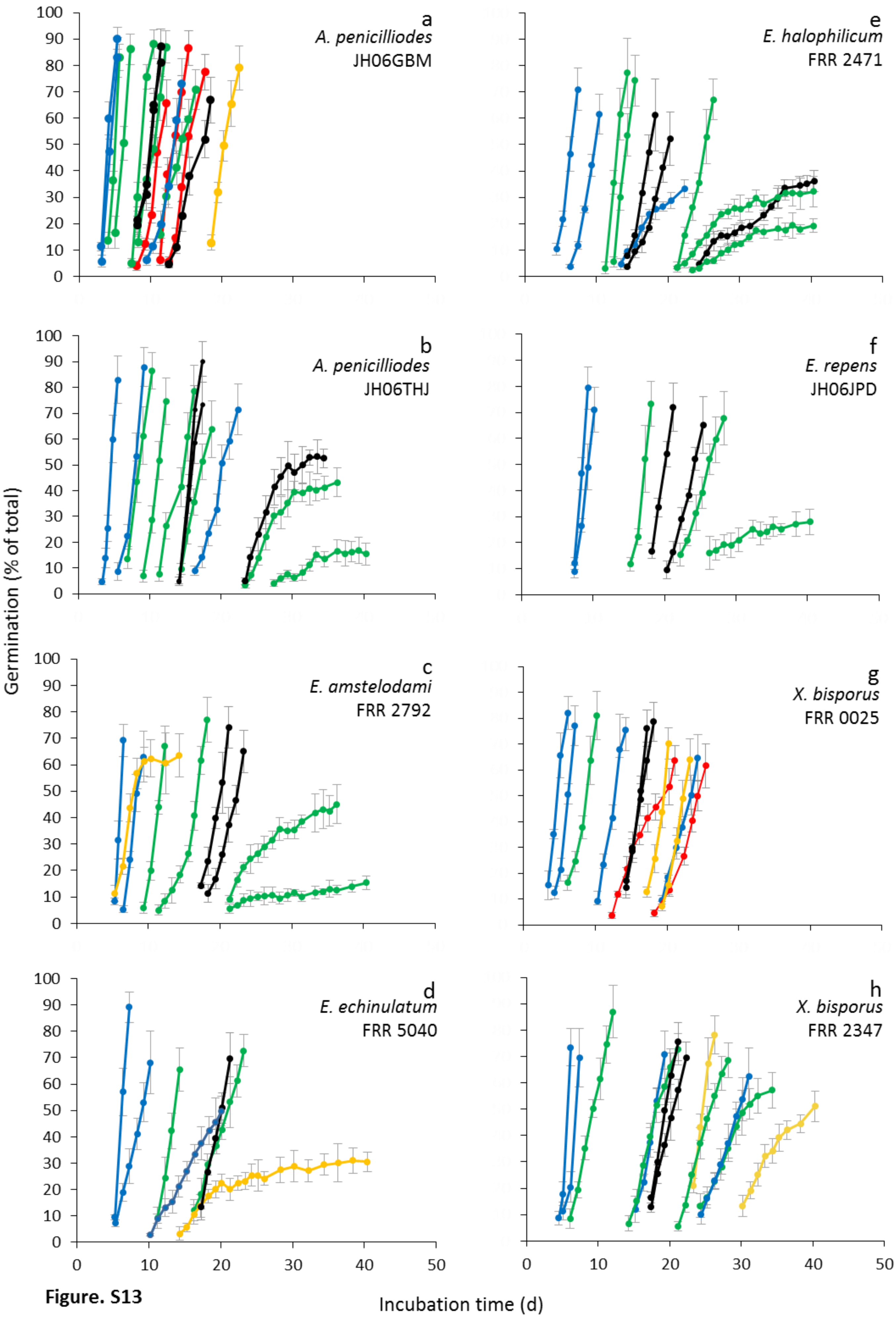
a
A. penicilliodes
JH06GBM
b
A. penicilliodes
JH06THJ
c
E. amstelodami
FRR 2792
d
E. echinulatum
FRR 5040
e
E. halophilicum
FRR 2471
f
E. repens
JH06JPD
g
X. bisporus
FRR 0025
h
X. bisporus
FRR 2347
Germination (% of total)
Incubation time (d)
0
10
20
30
40
50
60
70
80
90
100

Figure. S13

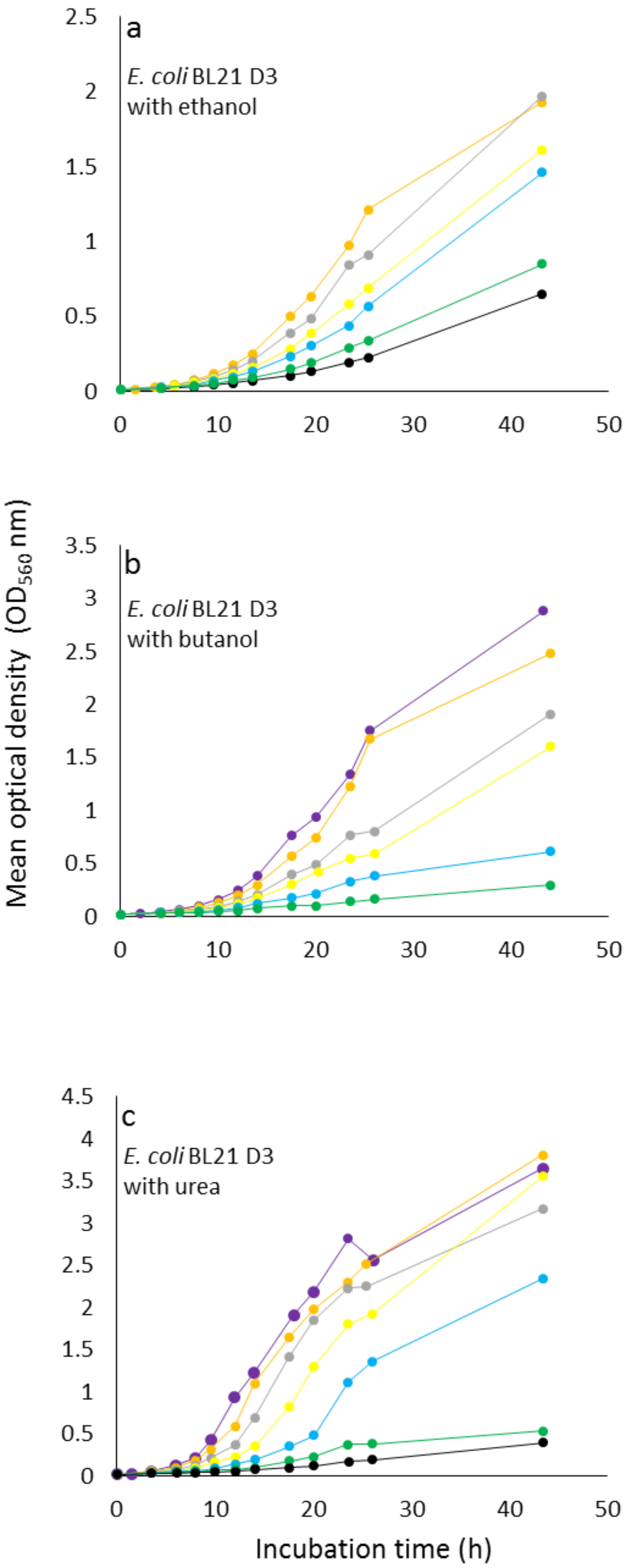
a
E. coli BL21 D3
with ethanol
b
E. coli BL21 D3
with butanol
c
E. coli BL21 D3
with urea
Mean optical density (OD560 nm)
Incubation time (h)
0
10
20
30
40
50
0
0.5
1
1.5
2
2.5
3
3.5
4
4.5

Figure. S14
